# Construction of iron-mineralized black phosphorene nanosheet to combinate chemodynamic therapy and photothermal therapy

**DOI:** 10.1080/10717544.2022.2039810

**Published:** 2022-02-17

**Authors:** Zhaoqing Shi, Jing Tang, Chuchu Lin, Ting Chen, Fan Zhang, Yuxing Huang, Ping Luan, Zhuo Xin, Qianqian Li, Lin Mei

**Affiliations:** a School of Pharmaceutical Sciences (Shenzhen), Sun Yat-sen University, Shenzhen, China; b Tianjin Key Laboratory of Biomedical Materials, Key Laboratory of Biomaterials and Nanotechnology for Cancer Immunotherapy, Institute of Biomedical Engineering, Chinese Academy of Medical Sciences and Peking Union Medical College, Tianjin, China; c School of Material Science and Engineering and Institute for Advanced Study, Nanchang University, Nanchang, China; d Guangdong Second Provincial General Hospital and Health Science Center, Shenzhen University, Shenzhen, China; e Shenzhen Bay Laboratory, Shenzhen, China

**Keywords:** Black phosphorene, chemodynamic therapy, drug delivery systems, Fenton reaction, photothermal therapy

## Abstract

Chemodynamic therapy (CDT) by triggering Fenton reaction or Fenton-like reaction to generate hazardous hydroxyl radical (•OH), is a promising strategy to selectively inhibit tumors with higher H_2_O_2_ levels and relatively acidic microenvironment. Current Fe-based Fenton nanocatalysts mostly depend on slowly releasing iron ions from Fe or Fe oxide-based nanoparticles, which leads to a limited rate of Fenton reaction. Herein, we employed black phosphorene nanosheets (BPNS), a biocompatible and biodegradable photothermal material, to develop iron-mineralized black phosphorene nanosheet (BPFe) by *in situ* deposition method for chemodynamic and photothermal combination cancer therapy. This study demonstrated that the BPFe could selectively increase cytotoxic ·OH in tumor cells whereas having no influence on normal cells. The IC_50_ of BPFe for tested tumor cells was about 3–6 μg/mL, which was at least one order of magnitude lower than previous Fe-based Fenton nanocatalysts. The low H_2_O_2_ level in normal mammalian cells guaranteed the rare cytotoxicity of BPFe. Moreover, the combination of photothermal therapy (PTT) with CDT based on BPFe was proved to kill tumors more potently with spatiotemporal accuracy, which exhibited excellent anti-tumor effects in xenografted MCF-7 tumor mice models.

## 1. Introduction

Over-production of H_2_O_2_ is a typical character of tumors, especially for solid malignance, which provides a promising biomarker to design selective anti-tumor therapeutics (Hanahan & Weinberg, 2011; Saravanakumar et al., 2017; Shi et al., 2020; Tang et al., 2021). In Fenton or Fenton-like reactions, H_2_O_2_ is catalyzed to hydroxyl radical (•OH), which is the most hazardous oxidative radical with the oxidative potential of 2.73V (Trachootham et al., 2009; Liu et al., 2019). Chemodynamic therapy (CDT) based Fenton or Fenton-like reactions have the potential to selectively kill tumors with high-level H_2_O_2_ without the need of O_2_, which has advantages in treating solid tumors with hypoxic microenvironment (Trachootham et al., 2009; Lin et al., 2018). Recently, transition metals like iron (Zhang et al., 2016; Chen et al., 2020; Pan et al., 2020a), copper (Li et al., 2019; Wu et al., 2019) and manganese (Lin et al., 2018; Ding et al., 2020) have shown talent in inducing Fenton reactions to realize CDT.

Iron-based agents are the most wildly employed materials as CDT agents among various transition metals because iron ions presented the highest Fenton catalytic efficiency (Zhang et al., 2016; Li et al., 2019; Chen et al., 2020). Currently, iron nano precipitates, iron oxide nanoparticles and iron mineral nanoparticles are developed to Fe-based CDT nano-systems (Huo et al., 2017; Liu et al., 2018; Chen et al., 2019; Fan et al., 2019; Lei et al., 2019; Pan et al., 2020a). These systems could degrade and release Fe ions in a tumor acidic environment. However, the amount of generated Fe ion species and the concentration of intracellular H_2_O_2_ are too low to induce a strong Fenton reaction (Dong et al., 2019; Wang et al., 2019). To solve these problems, Shi *et al.* designed NIR responsive iron oxide nanoparticles for enhancing the release of Fe ions, which achieved higher catalytic efficiency (Hu et al., 2017; Feng et al., 2019). Yeh *et al.* developed an H_2_O_2_-loaded Fe_3_O_4_@PLGA nano-system for enhancing CDT efficacy (Li et al., 2016) because the H_2_O_2_ could accelerate the release of Fe ions from iron oxides while providing substrates of Fenton reaction. These strategies improved the Fenton reaction efficacy by applying exogenous stimulation or increasing intracellular H_2_O_2_ concentration (Wang et al., 2020, 2021). More recently, Bu *et al.* synthesized amorphous Fe-based CDT agents for increasing the release rate of Fe ions, which showed enhanced Fenton reaction efficacy without exogenous stimulation (Chen et al., 2020). Therefore, it is evident that the Fe ions released from Fe-based nano-systems significantly determine the Fenton reaction efficacy (Hu et al., 2017). Compared with traditional iron oxide nanoparticles and iron mineral nanoparticles, novel nano-systems that are able to efficiently deliver and release Fe ions specifically at tumor sites should be developed.

Black phosphorene (BP), a kind of two-dimensional material, is a popular drug carrier for precise drug delivery because of its excellent photothermal ability, drug loading capacity and considerable biodegradability (Sun et al., 2015; Luo et al., 2019; Hu et al., 2020; Shi et al., 2020). BP is negatively charged, and it owns a high specific surface area, which can load drugs *via* electrostatic attraction and π – π stack (Wang et al., 2015; Liu et al., 2019; Luo et al., 2019; 2019; Shi et al., 2020). Therefore, decorating positive charged cargos or drugs with aromatic structures on the surface of BP is widely applied (Tao et al., 2017; Zeng et al., 2018; Pan et al., 2020b; An et al., 2021). Recently, Yu *et al.* demonstrated an *in situ* mineralization for loading Ca^2+^ on BPNS, and such method gained enhanced ion loading capacity than the previous electrostatic attraction method. Moreover, BP is a biodegradable photothermal agent, which could be used for efficient photothermal therapy (PTT) and photothermal on-demand drug release (Kuntz et al., 2017; Tao et al., 2017; Qiu et al., 2018; Zeng et al., 2018; Li et al., 2019; Liang et al., 2019; Luo et al., 2020).

**Figure 1.**
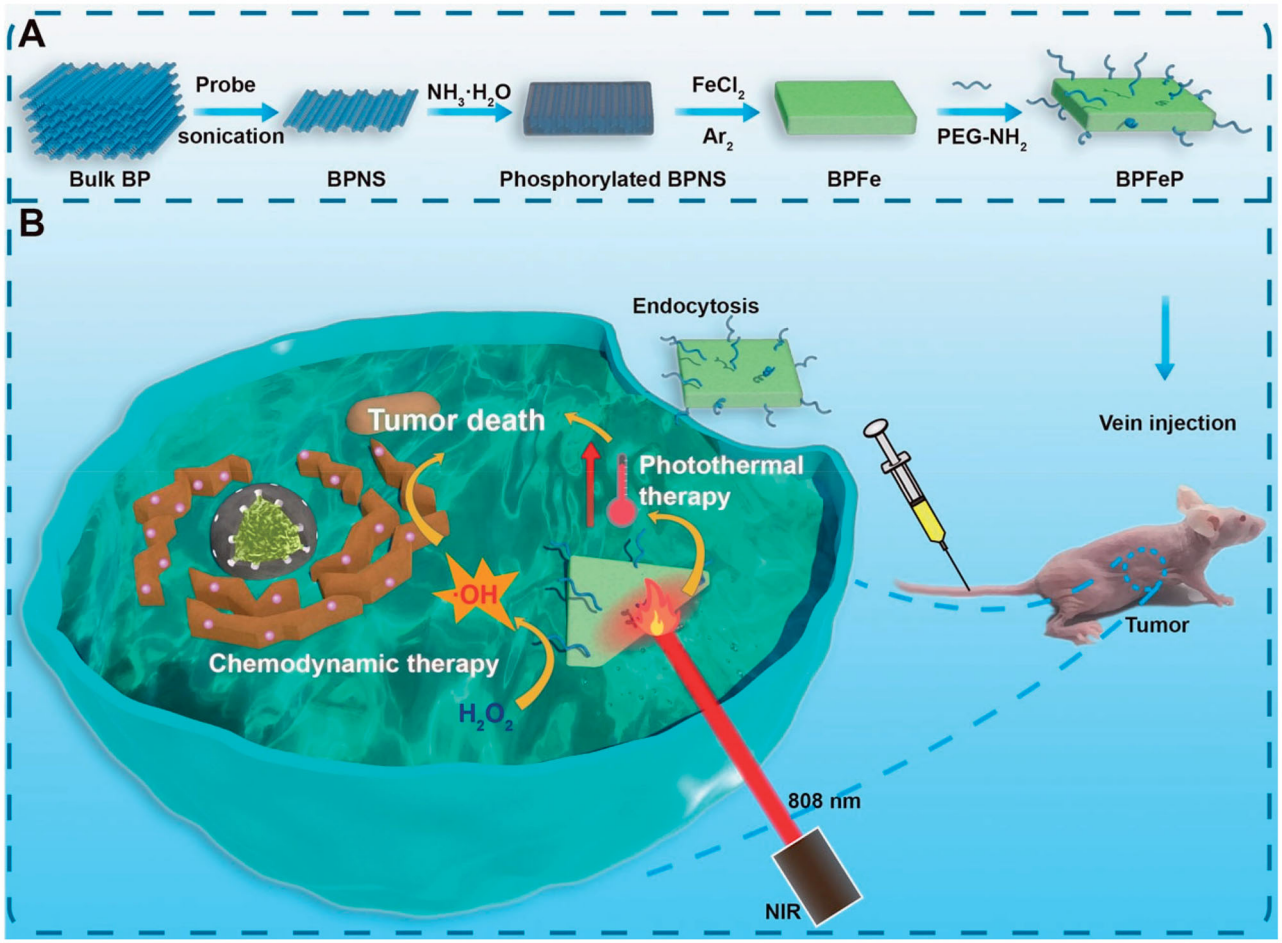
Schematic illustration of iron-mineralized black phosphorene (BPFe) for efficiently generating intracellular Fenton-reaction as an anti-tumor platform. (a) Preparation of BPFe: the bulk BP was exfoliated by probe sonication to obtain BPNS, and the FeCl_2_ was mineralized on ammonia-phosphorylated BPNS to prepare BPFe, which was finally PEGylated to give BPFeP. (b) Mechanism of BPFeP for chemodynamic and photothermal combination therapy.

Considering the favorable characteristics of BP for loading cations, we rationally designed a kind of iron-mineralized black phosphorene nanosheets (BPFe) to deliver and on-demand release Fe ions to tumor sites, which achieved CDT and PTT combination cancer therapy (Figure 1). Compared with previous iron oxide-based nano-systems, the BPFe showed better Fenton catalytic efficacy because the loaded Fe ion species could be rapidly released at a tumor acidic environment. Owing to this unique property, our BPFe showed an excellent anti-tumor effect in MCF-7 xenograft mice models only relying on the overproduced H_2_O_2_ of tumor cells. Besides, BPFe exhibited good safety on normal cells and major organs with a lower level of H_2_O_2_ than that of tumor cells. Moreover, the BPFe preserved the excellent photothermal properties of BPNS, which could combine CDT and PTT with spatiotemporal accuracy.

## 2. Experimental section

### 2.1. Materials

Bulk black phosphorus crystal (purity > 99.9%) was purchased from Nanjing Muke Nanotechnology (Nanjing, China). Iron (II) chloride and aqueous ammonia were purchased from Shanghai Macklin Biochemical Co., Ltd (Shanghai, China). Doxorubicin hydrochloride, methylene blue and N-methylpyrrolidone (NMP) were purchased from Aladdin (Shanghai, China). DMEM-H, RPMI-1640, fetal bovine serum (FBS), phosphate buffer saline of pH 7.2–7.4 (PBS), trypsin-EDTA, the penicillin-streptomycin solution were purchased from Gibco, Thermo Fisher Scientific. Cell counting kit-8 (CCK-8) was purchased from APExBio Technology (Shanghai, China). H_2_O_2_ assay kit (S0038) and ROS assay kit (S0033) were purchased from Beyotime Biotechnology (Shanghai, China). Fe_3_O_4_@PEI nanoparticle was obtained from Xi’an Ruixi Biological Technology (Xi’an, China). Annexin V-FITC/PI apoptosis detection kit and Calcein-AM live-cell staining kit were purchased from Solarbio Life Sciences (Beijing, China).

### 2.2. Preparation of BPNS, BPFe and BPFeP

Our BPFe was prepared by means of top to bottom strategy (shown in Figure 1). The protocol required 3 steps: exfoliation of bulk BP, phosphorylation of BPNS and *in situ* mineralization of Fe ions. The exfoliation of BP was to obtain size uniformed BPNS, and the detailed method was referred to in our previous research (Zeng et al., 2018). The size uniformed BPNS was then monodispersed in NMP and the mixture was added with NH_3_·H_2_O for phosphorylation, which was to transform the elemental phosphorus to negatively charged phosphate groups on BPNS surface (Pan et al., 2020b). Due to the strong association of phosphate with Fe ions, the Fe ions were simultaneously mineralized on the phosphate groups by gently adding FeCl_2_ to produce BPFe.

BPNS was prepared by the sonication exfoliation method (Zeng et al., 2018). 100 mg powder of ground bulk BP crystal was dispersed in 80 mL of NMP and sonicated with a Φ = 6 mm probe for 12 h in an ice bath under 650 W. Thereafter, the solution was then sonicated for 8 h under 600 W. Finally, BPNS with uniformed size was obtained by gradient centrifugation. The solution was centrifuged at 7000 rpm for 15 min to isolate large bulks, and the supernatant was collected for further centrifugation at 14,000 rpm for 20 min to obtain the BPNS precipitates.

BPFe was prepared by *in situ* mineralization method, which was detailly shown in Figure 1a (Pan et al., 2020b). 5 mg of BPNS was dispersed in 100 mL of NMP (50 μg/mL) and sonicated in the water bath to form a uniform dispersion, and then 100 μL 28% aqueous ammonia was added to the solution to phosphorylate the surface of BPNS. The mixture was vigorously stirred at 37 °C for 2 h. To deposit the iron ions on the surface of BPNS, 5 mL FeCl_2_/ethanol solution with a feeding ratio of 1:20 was gently added into the mixture under the protection of argon for 12 h. Finally, the BPFe was collected by centrifugation and washed with ethanol several times. The as-prepared BPFe was vacuum dried or stored in ethanol for further use.

BPFeP was prepared by the PEGylation method according to our previous research (Tao et al., 2017). In brief, 2 mg BPFe was dispersed in 1 mg/mL PEG-NH_2_ aqueous solution, and the mixture was sonicated for 0.5 h *via* probe sonication. Then the mixture was stirred for another 3 h and the BPFeP was obtained by centrifugation.

### 2.3. Characterization

Transmission electron microscope (TEM) images of BPNS and BPFe were performed on JEM-1400 to observe the morphology. EDS mapping was performed on JEM-F200 to observe the elemental distribution. Atom force microscopy (AFM) was employed to determine the thickness of nanosheets, which was measured by Nanoscope IIId. Dynamic light scattering (DLS) and phase analysis scattering (PALS) were analyzed on Brookhaven NanoBrook 90Plus PALS to determine the hydrodynamic size and zeta potential of BPNS, BPFe and BPFeP.

XPS survey was tested by ESCALAB 250Xi to analyze the surface composition of BPFe. ICP-MS was employed for quantification of BPFe, and the UV-vis spectrophotometry was determined by PerkinElmer LAMBDA 365 to analyze optical properties and study the linear relationship between concentration and absorption. The photothermal property was conducted by an 808 nm diode laser fiber coupling system and Fluke Ti450 thermal imaging camera.

### 2.4. Cell culture

Tumor and normal cell lines were used in this study: MCF-7, human breast cancer cells; Hep1-6, murine hepatoma cells; B16-F10, murine melanoma cells; NCM460, normal human colon mucosal epithelial cells. All the cells were purchased from CTCC and cultured by 89% DMEM-H (or RPMI-1640) + 10% FBS + 1% P/S in a 5% CO_2_ balanced incubator at 37 °C.

### 2.5. Preparation of DOX-labeled BPFe and cellular uptake evaluation

To evaluate the cellular uptake of BPFe, we employed DOX as a fluorescent label for flow cytometry (FCM, Beckman Coulter CytoFLEX) and confocal laser scanning microscopy (ZEISS LSN880). To label DOX on the BPFe, 1 mg BPNS were dispersed in 20 mL NMP, and 0.1% aqueous ammonia was added. After 2 h, the DOX with a feeding ratio of 1:3 was carefully added into the mixture and stirred for 12 h to obtain BPFe@DOX. The BPFe@DOX was collected by centrifugation and washed several times.

The FCM was used to quantitatively determine the cellular uptake behavior, 10^5^ cells were incubated in a 6-wells plate and treated with 20 μg/mL BPFe@DOX at different time points. Finally, the cells were collected for FCM detection on the PC5.5 channel. The data was processed by CytExpert. The confocal laser scanning microscope (CLSM) was used to qualitatively observe the uptake of BPFe. The cells were seeded in the glass-bottom dish, and the incubated cells were observed by CLSM with a 488 nm laser.

### 2.6. Intracellular H_2_O_2_ concentration determination

The intracellular H_2_O_2_ concentration of tumor and normal cell lines was determined by the H_2_O_2_ assay kit. Briefly, the tumor and normal cell lines were cultured on a large scale and 10^7^ cells were harvested for determination. The cells were washed with cold PBS twice, and then lysis solution was added to release intracellular H_2_O_2_. Finally, the mixtures were centrifuged at 14,000 *g* at 4 °C, and the supernatants were collected for determination according to the H_2_O_2_ assay kit.

### 2.7. Hydroxyl radical determination

The hydroxyl radical (⋅OH) was determined by methylene blue (MB), a hydroxyl radical capturing reagent (the MB could capture the produced ⋅OH and then fade at the wavelength of 640 nm). Hence the amount of ⋅OH was correlated to the absorbance reduction of MB. Briefly, 0.5 mL of different samples were added into 1 mL of 2 mg/mL MB solution, and then 0.5 mL of 200 μM H_2_O_2_ was added to initiate the determination reaction. The absorbance of each measurement was determined by a UV-vis spectrophotometry.

### 2.8. Cellular ROS assays

The ROS assays were performed by ROS assay kits provided by Beyotime. Briefly, the cells were seeded in a 6-wells plate with a density of 10^6^ cells per well. The cells were treated with different drugs for 2 h and then loaded with DCFH-DA for 30 min. The cells were washed with cold PBS, and FCM or CLSM experiments were applied immediately. FITC channel was chosen for FCM and DCF channel was chosen for CLSM.

### 2.9. Cytotoxicity assay

The cytotoxicity assays were performed *via* the CCK-8 method according to the manufacturer’s protocol. Briefly, the cells were counted and seeded into a 96-wells plate with a density of 5000 cells/well. After that, the plate was incubated for 12 h, and the medium was replaced with the drug-containing medium of indicated concentration respectively (6 parallel wells) and incubated for 24 h. Finally, 10 μL of CCK-8 solution was added to each well and incubated for 2 h. The optical absorption at 450 nm of the plate was determined by a microplate reader (SpectraMax i3x).

### 2.10. Photothermal properties characterization

The photothermal properties characterizations were referred to in our previous research (Zeng et al., 2018). Briefly, 1 mL of the sample with indicated concentrations was added into 1 cm × 1 cm quartz cells, and the 808 nm NIR laser (dot size = 1 cm^2^, power = 1 W) was irradiated on the samples vertically. A thermal camera (Fluke) was employed to record the temperature change against time.

### 2.11. *In vitro* photothermal therapy study

Cytotoxicity assays and CLSM were employed to evaluate the photothermal anti-tumor effect of BPFe on MCF-7. For cytotoxicity assays, the cells were seeded in a 96-wells plate with a density of 5000 cells per well. The BPFe with indicated concentration was added to wells and incubated for 4 h. Then the plate was irradiated by 808 nm NIR laser (1 W, 5 min per well) and incubated for another 20 h and finally evaluated by CCK-8 assays. For CLSM observation, 2 × 10^5^ cells were seeded in a glass-bottom dish and cultured for 24 h. The BPFe was then administrated and incubated for 4 h. To observe the laser on-off effect, we employed a pierced foil to control the irradiated area, and the cells were irradiated by NIR laser at 1 W cm^−1^ for 5 min at the selected area. The cells were incubated for another 6 h and treated with Calcein-AM to detect alive cells under the observation of CLSM.

### 2.12. *In vitro* photothermal and chemodynamic therapeutic effect of BPFeP

To evaluate whether therapeutic activities of PEGylated BPFe (BPFeP) were similar to BPFe, we had performed a cytotoxicity assay and cellular ROS assay to investigate the photothermal and chemodynamic therapeutic effect of BPFeP. The protocols and conditions were according to previous assays.

### 2.13. Hemolysis test

A hemolysis test was performed to investigate the safety and biocompatibility of the nano-system. In brief, fresh blood was obtained from SD rats, and then it was centrifuged at 2000 rpm for 10 min to obtain red blood cells. The red blood cells were gently washed by PBS 3–5 times and then diluted by PBS (1:10). 200 μL diluted red blood cells were then gently added into 800 μL of test solution. The mixture was incubated for 1 h at 37 °C and centrifuged at 1000 rpm to isolate unbroken RBCs, and the optical density of supernatants was analyzed at 570 nm.

### 2.14. Xenograft tumor models

The assays of animal experiments were under the guidance of the Administrative Committee on Animal Research in Sun Yat-sen University. To establish xenograft models, 0.1 mL of MCF-7 cell suspensions in PBS (2 × 10^6^ cells for each mouse) were subcutaneously injected to the oxter side to establish tumors.

### 2.15. *In vivo* photothermal therapy

The MCF-7 tumor-bearing mice with a tumor volume of around 300 mm^3^ were intravenously injected with 200 μL of PBS, BPFe and BPFeP, respectively. After 12 h administration, the mice were anesthetized and irradiated by 808 nm NIR laser with a power of 1.5 W cm^−1^ for 5 min. The tumor temperature was monitored instantly *via* the photothermal imaging camera to determine the therapeutic effect and avoid overtreatment.

### 2.16. *In vivo* anti-tumor effect and biosafety examination

The MCF-7 tumor-bearing mice were randomly grouped when the average tumor size reached 100 mm^3^. The Control group was intravenously injected with 200 μL of PBS; NIR group was intravenously injected with 200 μL of PBS and treated with 808 nm NIR laser for 5 min after administration; BPFeP group was intravenously injected with 200 μL of BPFeP; BPFeP + NIR group was intravenously injected with 200 μL of BPFeP and treated with 808 nm NIR laser for 5 min after administration. All the groups were administrated with indicated drugs on days 1, 3 and 7, and the BPFeP group was treated with NIR on days 4 and 8 (12 h after each administration).

The tumor volumes and body weights were recorded every two days to examine the anti-tumor effect of each group. The tumor volumes (V) were calculated as V = Length × Width × Width/2. The tumor tissues and major organs of the participated mice were harvested for further observation. A picture of the excised tumors was taken. The major organs were then fixed in 4% paraformaldehyde for further H&E staining.

### 2.17. Statistical methods

The experimental data were presented as mean ± standard deviation. Statistical analysis was performed *via* one-way ANOVA on Origin Pro 2021 (OriginLab Corporation). Different statistical significance was presented as *p* < .05 (*), *p* < .01 (**) and *p* < .001 (***).

## 3. Results and discussion

### 3.1. Characterizations of BPFe

**Figure 2.**
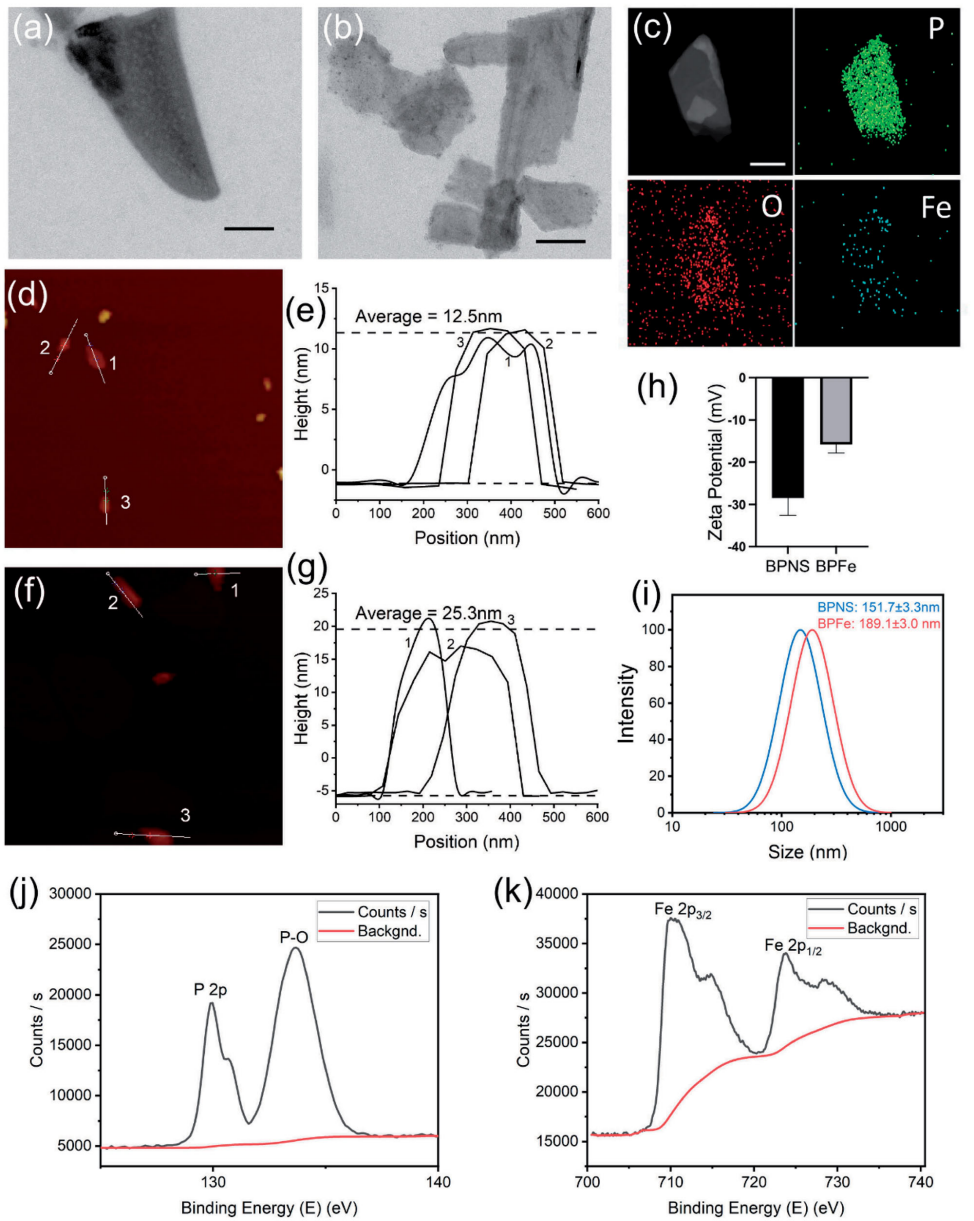
Characterization of BPFe. (a) TEM image of BPNS (scale bar = 100 nm); (b) TEM image of BPFe (scale bar = 100 nm); (c) EDS mapping images of BPFe (scale bar = 100 nm); AFM image (d) and height analysis (e) of BPNS; AFM image (f) and height analysis (g) of BPFe; (h) Zeta potential of BPNS and BPFe; (i) Particle size distributions of BPNS and BPFe; (j) P 2p scan of XPS survey on BPFe; (k) Fe 2p scan of XPS survey on BPFe.

To prove the successful preparation of BPFe, we firstly observed the BPNS and BPFe *via* transmission electron microscope (TEM) (Figure 2a,b). TEM images have shown that both materials were two-dimensional sheet structures. EDS mapping images (Figure 2c) had proved that the Fe and O elements were distributed on the nanosheets, which indicated the success of BPNS phosphorylation and Fe ion decoration. We next measured the thickness of BPNS and BPFe by atom force microscopy (AFM) (Figure 2d,f). According to the height information (Figure 2e,g), the average thickness of BPFe was 25.3 nm, which was higher than that of BPNS (12.5 nm). AFM experiments illustrated that deposition of Fe ions could increase the thickness of nanosheets. Then we determined the zeta potential and hydrodynamic particle size of the BPNS and BPFe. Zeta potential (Figure 2h) revealed that the negative charge potential of BPNS was reduced since depositing Fe ions. Dynamic light scattering (DLS) (Figure 2i) showed that the effective size of BPNS and BPFe was 151.7 ± 3.3 nm and 189.1 ± 3.0 nm, and the PDI were 0.238 and 0.227, respectively. The increase in the particle size also indicated the successful preparation of BPFe.

We further performed XPS to study the surface composition of BPFe. P 2p scan of BPFe (Figure 2j) revealed the presence of P-O bond, a phosphorated form of P. Fe 2p scan of BPFe (Figure 2k) showed the presence of Fe^2+^ and Fe^3+^, and the atomic ratio between Fe^2+^ and Fe^3+^ was calculated as 1.67. Therefore, the Fe ions have mainly existed as iron (II) phosphates on the surface of BPNS. Thorough atomic ratio analysis of XPS was given in Table S1. Besides, the Fe to P mass ratio of BPFe was 1.041 determined by ICP-MS, which indicated that such *in situ* mineralization method could effectively load Fe ions. The prepared sample and optical absorption spectra were shown in Figure S1, and the linear relationship of BPFe between concentration and absorption was shown in Figure S2. All these results indicated that we successfully prepared BPFe by *in situ* mineralization. Moreover, we found that the BPFe had faster degradation under acidic pH of 6.0 than pH of 7.0 which indicated that the BPFe could respond to tumor acidic environment and degrade to release Fe ions (Figure S3).

**Figure 3.**
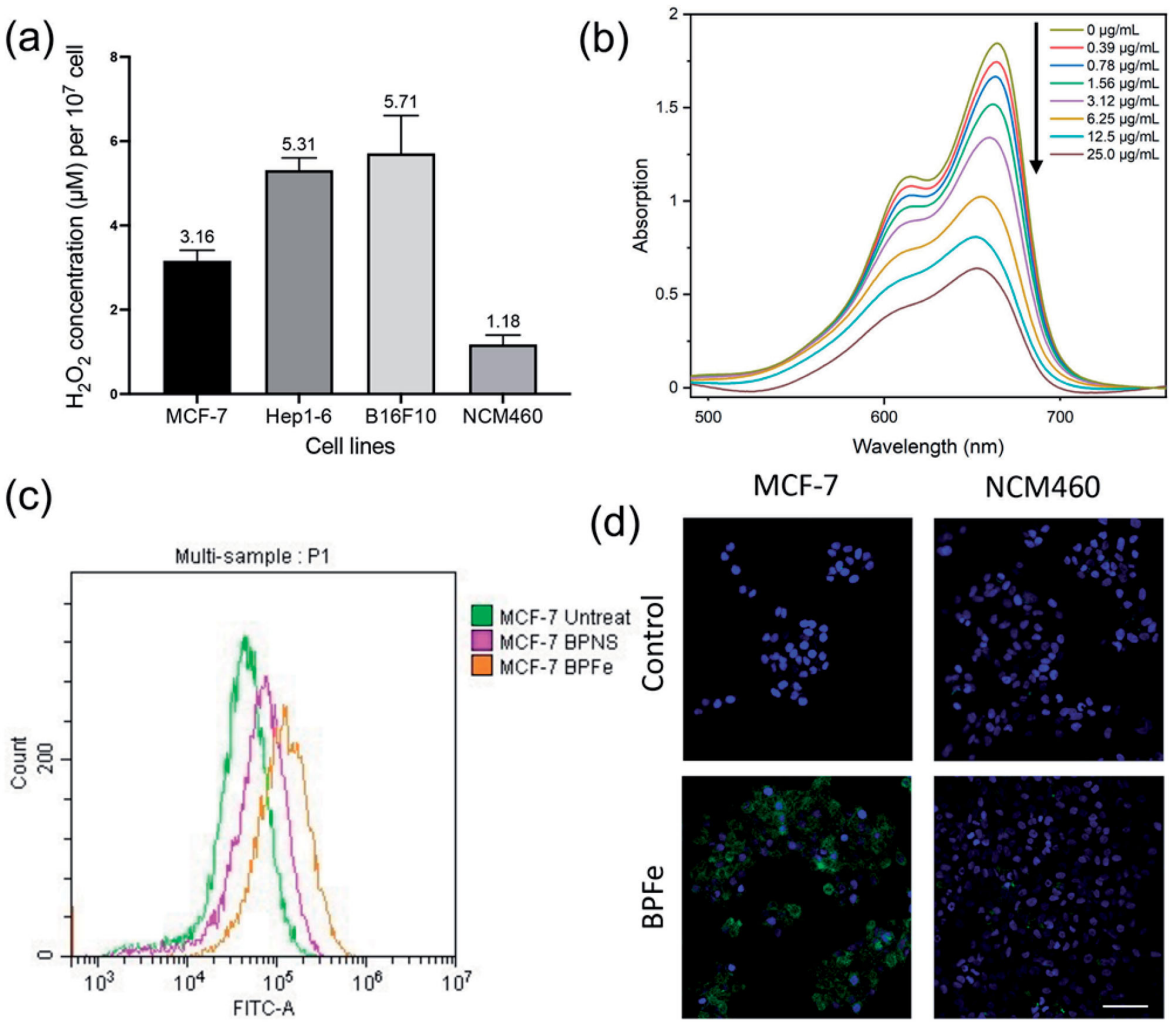
*In vitro* ROS and •OH assays. The BPFe could generate more •OH in tumor cells with higher H_2_O_2_ levels compared to normal mammalian cells. (a) H_2_O_2_ concentration (per 10^7^ cells) of selected cell lines. (b) The absorbance of MB after treatment with the indicated concentration of BPFe. (c)FCM analysis of MCF-7 ROS level after treated with BPNS or BPFe. (d) Confocal images of DCFH fluorescence intensity of MCF-7 and NCM460 (Blue channel: DAPI, green channel: DCFH, scale bar = 100 μm).

### 3.2. Cell uptake of BPFe

In order to examine cellular uptake of BPFe, we labeled doxorubicin (DOX) on BPFe for its easy stack on BP and stable fluorescence even after loading. 4T1 and MCF-7 cell lines were chosen to examine the uptake efficiency. CLSM images (Figure S4a) indicated that the ingested BPFe was distributed in the cytosol. FCM analysis (Figure S4b) showed the proportional relationship between time and uptake amount. As a result, the BPFe could be internalized by the tumor cells efficiently.

**Figure 4.**
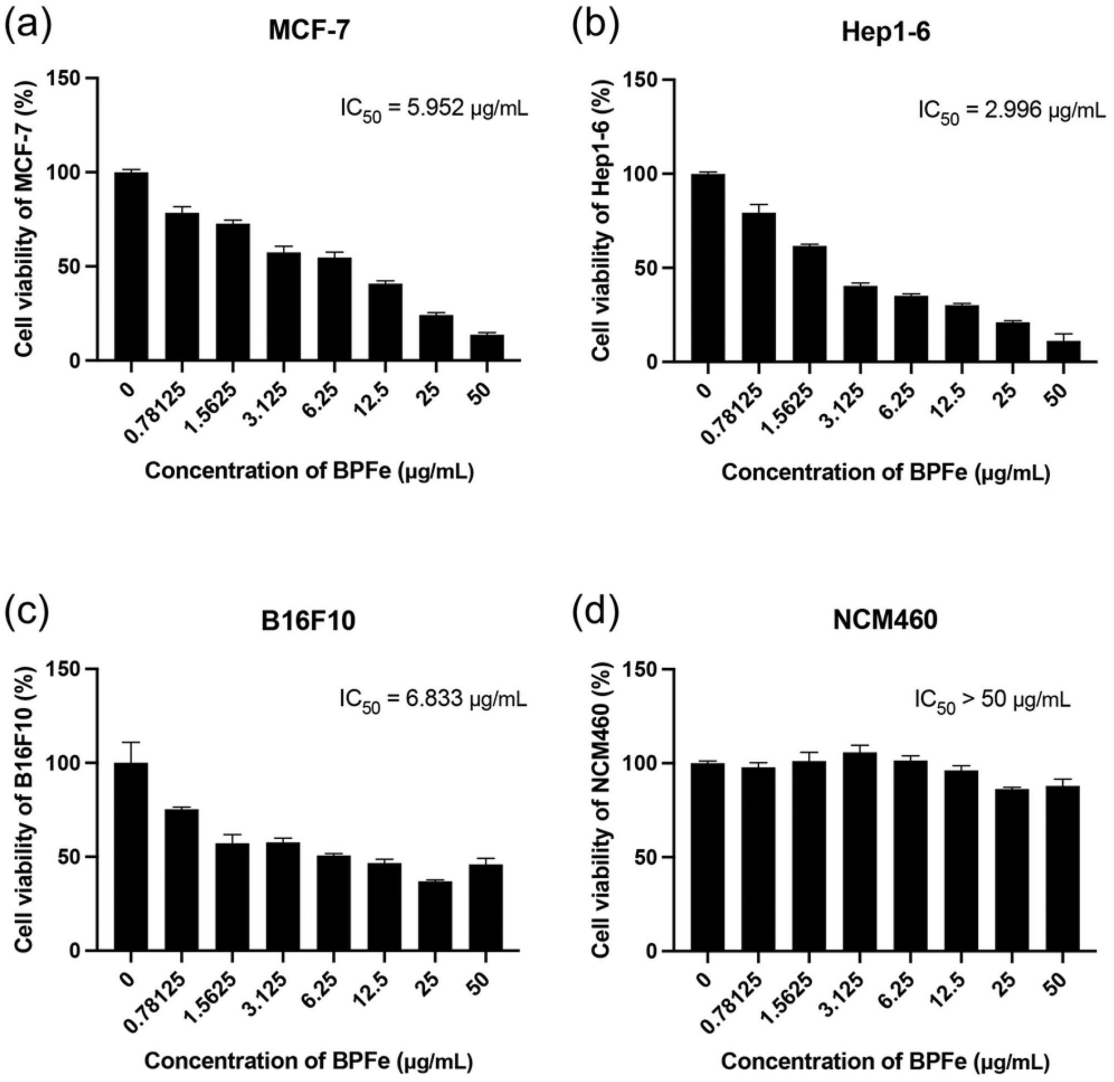
Cytotoxicity assays of BPFe. Tumor cell lines: (a) MCF-7, (b) Hep 1–6, (c) B16F10. Normal cell line: (d) NCM460.

### 3.3. Hydroxyl radical production mediated by BPFe

The theoretical basis that BPFe could selectively inhibit tumor cells was the H_2_O_2_ concentration difference between tumor and normal cells. Hence, we first validated the intracellular H_2_O_2_ concentration difference between tumor (MCF-7, Hep1-6 and B16F10) and normal (NCM460) cell lines (Figure 3a). The results indicated that such a concentration difference did exist. To evaluate the ·OH production of BPFe *in vitro*, MB was employed to detect the ·OH. The decreased absorbance of MB (Figure 3b) manifested that the BPFe could catalyze H_2_O_2_ to produce ·OH in a concentration-dependent manner. Moreover, our BPFe showed better ·OH production than previously published Fe_3_O_4_ nanoparticles (as shown in Figure S5), which might derive from the faster release of Fe ions of BPFe than Fe_3_O_4_. We also compared the ·OH production ability of bare BPNS with BPFe. The results (Figure S6a,b) demonstrated that BPFe generated much more ·OH than BPNS, which indicated that the Fe deposition contributed to the production of ·OH.

**Figure 5.**
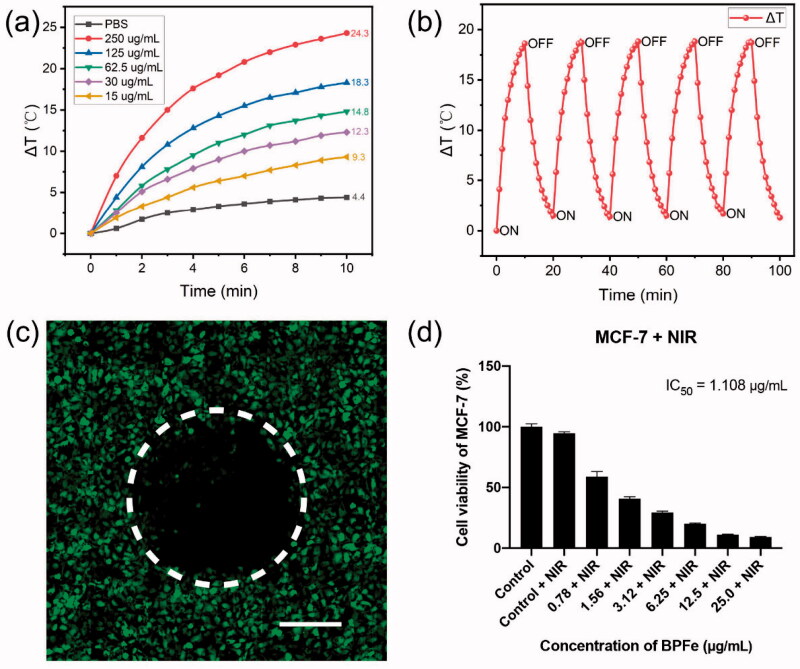
The photothermal properties of BPFe. (a) Temperature elevation curves of BPFe with different concentrations under 808 nm laser (1 W cm^−1^). (b) Photothermal stability of BPFe for five lasers on/off cycles under 808 nm laser (1 W cm^−1^). (c) CLSM images of MCF-7 after being treated with BPFe and NIR irradiation, the green fluorescence was Calcein-AM stained live cells (Scale bar = 200 μm). (d) Cell viability of MCF-7 after treatment with BPFe and NIR irradiation.

**Figure 6.**
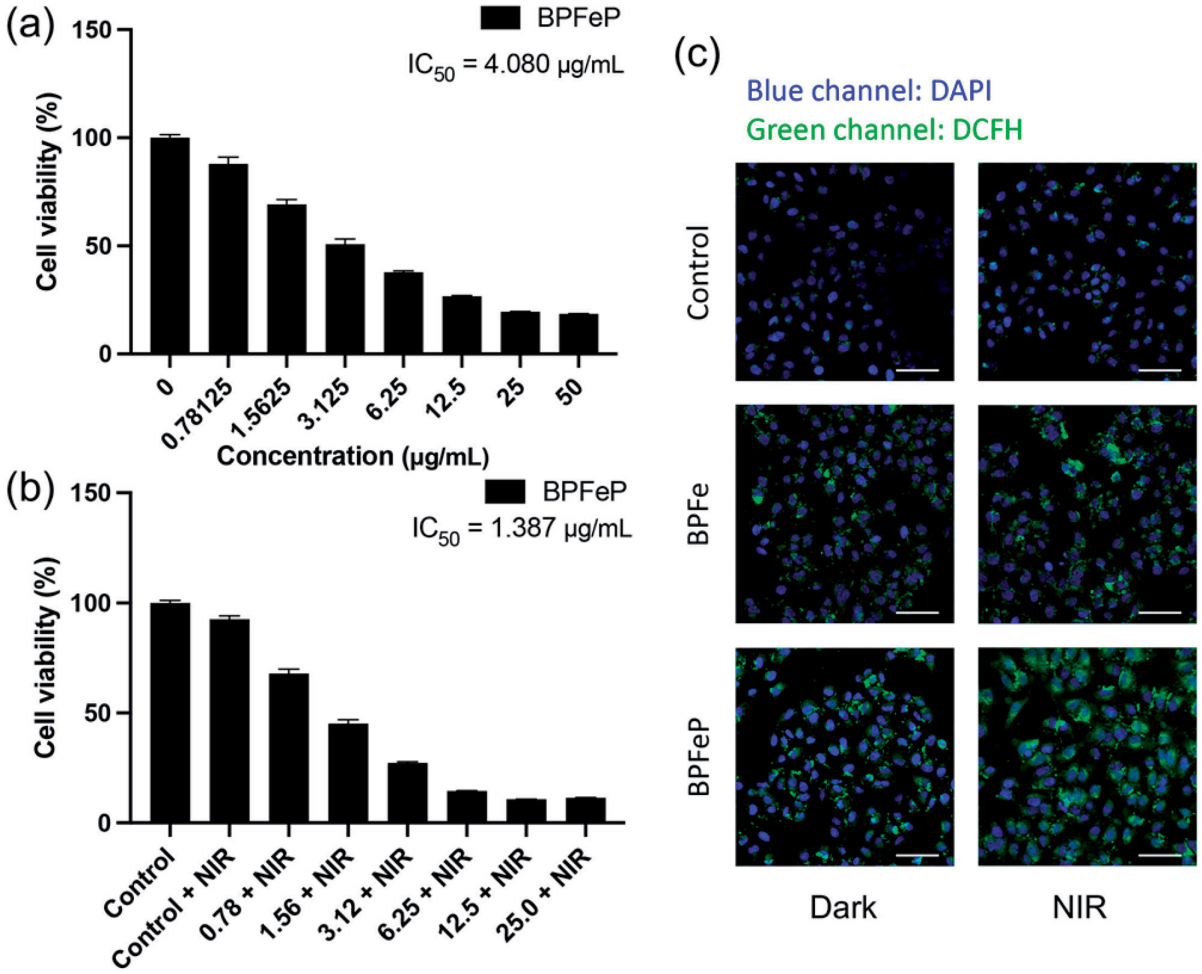
BPFeP showed similar therapeutic activities to BPFe. (a) Cell viability of MCF-7 after treatment with the indicated concentration of BPFeP. (b) Cell viability of MCF-7 after treatment with the indicated concentration of BPFeP and NIR irradiation (under 808 nm laser, 1 W cm^−1^, 5 min). (c) CLSM images of MCF-7 for observing intracellular ROS generation under different treatments (Dark group was not treated with NIR, NIR group was treated with 808 nm laser, 1 W cm^−1^, 5 min. Scale bar = 100 μm).

Then, we investigated the ROS levels in the cellular level *via* DCFH-DA, a ROS probe, after treatment with different drugs. Both FCM and CLSM analyses revealed that the BPFe increased the intracellular ROS in tumor cells while having a weak influence on normal cells. A representative example, MCF-7, was shown in Figure 3(c,d). Similar results were found in other tumor cells, like Hep1-6, B16F10 (Figure S7). Meanwhile, the ROS level remained low in normal cells after treatment of BPFe, like NCM460 (Figure 3d). These results indicated that BPFe was able to utilize the different levels of H_2_O_2_ between cancer and normal cells to selectively generate ·OH and increase the ROS stress in tumor cells.

**Figure 7.**
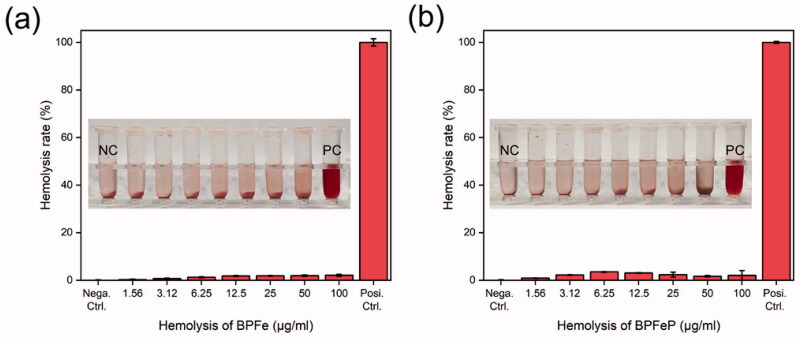
Hemolysis assays to (a) BPFe and (b) BPFeP. Tubes in the picture were listed as NC (negative control), drugs of indicated concentration and PC (positive control).

### 3.4. Cytotoxicity of BPFe

Bare BPNS was known for its good biocompatibility and safety, which did not show obvious toxicity in both tumor and normal cells (Figure S8). Unlike BPNS, BPFe showed relatively higher toxicity in tumor cells (IC_50_ from 2.996 μg/mL to 6.833 μg/mL, Figure 4(a–c) than normal cells (IC_50_ more than 50 μg/mL, Figure 4c). According to the determined H_2_O_2_ level (Figure 3a), we compared the relationship between H_2_O_2_ level and IC_50_. As shown in Figure S9, BPFe exhibited higher cytotoxicity in H_2_O_2_ overproduced tumor cells than normal cells, because it could induce Fenton reaction to generate more hazardous •OH in these tumor cells.

**Figure 8.**
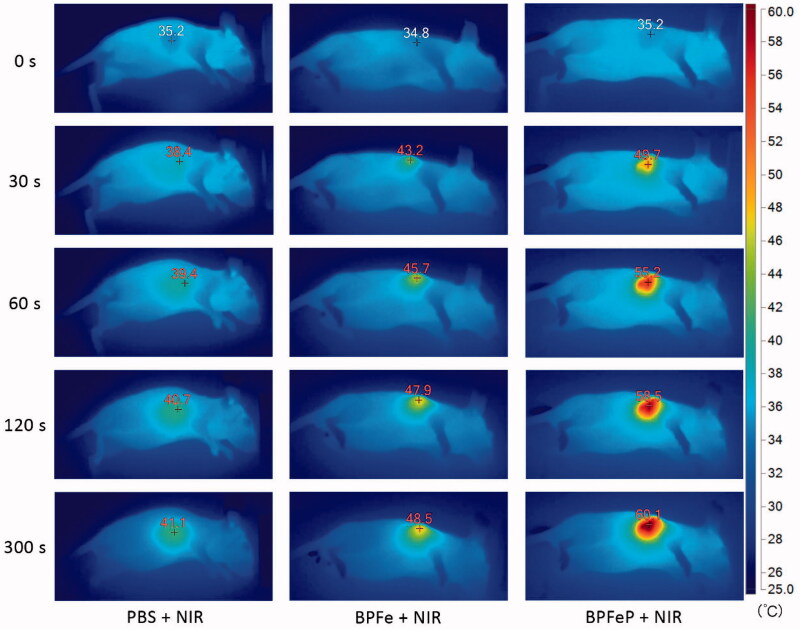
Photothermal images of MCF-7 tumor-bearing mice irradiated *via* 808 nm NIR laser after 12 h of drug administration.

**Figure 9.**
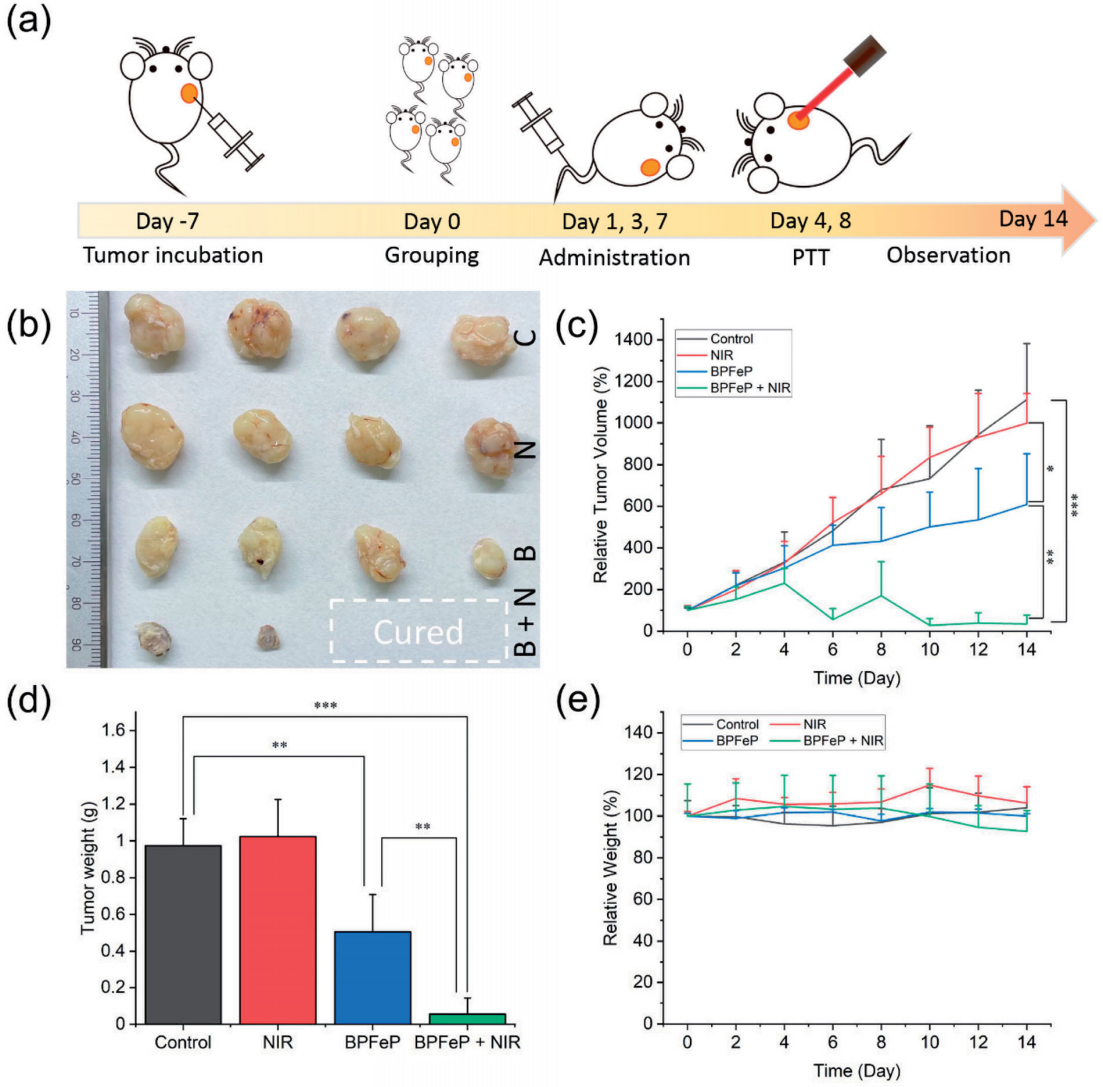
*In vivo* chemodynamic and photothermal synergistic treatment. (a) Schematic illustration of treatment procedures. (b) Excised tumors after 2 weeks of treatment. The C, N, B and B + N represented as Control, NIR, BPFeP and BPFeP + NIR, respectively. (c) Tumor volume of mice after indicated treatment. (d) Weight of excised tumor. (e) Mice body weight change. (*n* = 4, different *p* was marked as *, **, *** while *p* < .05, .01 and .001.).

Considering the ROS-level shown in Figure 3(a), we concluded that tumor cells were more sensitive to BPFe, because the Fenton reaction could cause strong oxidative stress in tumor cells. On the contrary, the normal cells could avoid such oxidative stress for their low H_2_O_2_ level.

### 3.5. In vitro photothermal therapy effect of BPFe

Other than its selectivity between tumor and normal cell lines, the BPFe also showed good photothermal activity. We examined the photothermal properties of BPFe under 808 nm NIR laser irradiation (1 W cm^−1^). After irradiation for 10 min, BPFe could increase 24.3 °C with the concentration of 250 μg/mL (Figure 5a), which was higher than that of BPNS (23.3 °C, as shown in Figure S10). Besides, we investigated the photothermal stability of BPFe: 125 μg/mL BPFe was irradiated under 808 nm NIR laser irradiation (1 W cm^−1^) for 10 min and placed for cooling. As shown in Figure 5(b), the BPFe could still rise back to the initial maximum temperature even after 5 cycles of irradiation.

We next examined the photothermal cytotoxicity on the MCF-7 cell line. To observe the photothermal on-off effect of BPFe, we partially irradiated the BPFe treated cells, and stained the live cells with Calcein-AM. Figure 5(c) obviously showed that rare cells were alive in the irradiated area. Besides, cytotoxicity assays had demonstrated that the photothermal treatment decreased the IC_50_ of BPFe by 5-fold (from 5.952 to 1.109 μg/mL) (Figure 5d). Moreover, we performed apoptosis analysis on MCF-7 to explore the cell death mechanism induced by BPFe. The results (Figure S11) demonstrated that BPFe could induce tumor cell apoptosis, and sole NIR irradiation treatment was nontoxic. As a result, BPFe showed an enhanced tumor-killing effect while combined with NIR irradiation.

### 3.6. *In vitro* photothermal and chemodynamic effect of BPFeP

PEGylation is a widely used surface modification in nano-system, which is proved to enhance biocompatibility and blood circulation behaviors. We employed PEG-NH_2_ to decorate on BPFe to prepare BPFeP, and hydrodynamic particle size and zeta potential of BPFeP were characterized. The results (Figure S12) showed that the particle size of BPFeP increased to 295.2 nm and the negative charge of BPFe decreased in BPFeP, which indicated the successful decoration of PEG. Besides, the BPFeP also showed similar photothermal properties with BPFe (Figure S13).

We performed cytotoxicity assays to determine the anti-tumor activities of BPFeP. The results (Figure 6a,b) demonstrated that the BPFeP had similar chemodynamic and photothermal effects to BPFe. Furthermore, we performed CLSM to determine ROS generation. Figure 6(c) demonstrated that the BPFeP could generate adequate intracellular ROS in dark, meanwhile, more ROS was detected after being treated with 808 nm NIR. As a result, the NIR treated BPFeP group showed the highest ROS level, which indicated that the BPFeP group could realize successful chemodynamic and photothermal combinations *in vitro*.

### 3.7. Hemolysis assays of BPFeP

We performed hemolysis assays to investigate the safety of our nano-system before *in vivo* study. The BPFe and BPFeP with concentrations of 1.56–100 μg/mL were mixed with red blood cells. After incubating for 1 hour at 37 °C, the samples were centrifuged to observe the hemolysis. As shown in Figure 7, both BPFe and BPFeP did not show obvious hemolysis compared with positive control. The hemolysis rates were calculated through the optical density of supernatants at 570 nm. All the hemolysis rates were below 5%, which indicated the safety of BPFeP.

### 3.8. *In vivo* photothermal imaging

Previous results confirmed that BPFeP had good biocompatibility and excellent photothermal properties *in vitro*. We further performed *in vivo* photothermal imaging assays to observe the photothermal effects of BPFeP. As shown in Figure 8, the PBS group increased up to 41.1 °C. The temperature of the BPFeP group increased to 60.1 °C, which was higher than that of BPFe (48.5 °C). This indicated that the PEGylation could endow the BPFe with the long-circulation ability and higher stability *in vivo*. Such a feature of BPFeP could increase the accumulation of BPFeP in the tumor site.

### 3.9. *In vivo* anti-tumor effect of BPFeP

We further tested the chemodynamic and photothermal synergistic treatment effects on tumor-bearing mice. The treatment procedures were shown in Figure 9(a): the MCF-7 xenograft tumor-bearing mice were administrated with saline or BPFeP at days 1, 3 and 7, and the 808 nm NIR irradiation was given at days 4 and 8 for NIR-involved groups. After 2 weeks of treatment, we excised the tumors from each group, which were compared in Figure 9(b), and found that there were two mice without a residual tumor in BPFeP + NIR group. The tumor weight is shown in Figure 9(d) manifested similar results. The tumor growth curve (Figure 9c) also demonstrated the anti-tumor effect of BPFeP. Although the tumors were not completely controlled after sole treatment of BPFeP, a combination of chemodynamic and photothermal therapy inhibited the tumor growth excellently. The body weight (Figure 9e) of the mice did not show obviously losing during the whole treatment. Moreover, the H&E staining results (Figure S14) showed no obvious lesion on major organs of all groups, which proved its good biocompatibility and safety.

## 4. Conclusions

Many differences in the tumor microenvironment allowed scientists to distinguish tumor tissues or cells from normal ones. Overproduction of H_2_O_2_, acidic extracellular environment and hypoxia condition are well-established biomarkers of tumors. The chemodynamic therapy that utilizes these features has become important antitumor therapeutics. However, the slow release of active Fe ions from traditional Fe-based CDT-inducers, like iron oxide nanoparticles and iron mineral nanoparticles, limited the outcome of CDT. Recently, an amorphous Fe-based nano-system showed fast release of Fe ions and excellent chemodynamic effect, which indicated the importance of developing a novel Fe-based CDT inducer. Herein, we rationally designed iron-mineralized black phosphorene nano-systems to gain BPFeP. BPFeP directly loaded individual Fe ions on BPNS and showed a much better Fenton effect than traditional Fe_3_O_4_ nanoparticles, which might owe to the easy release of Fe ions from BPFeP. BPFeP exhibited good biosafety and biocompatibility to normal mammalian cells. Meanwhile, BPFeP selectively killed tumor cells with higher H_2_O_2_ levels *in vitro*. Further experiments with tumor-bearing mice indicated that BPFeP selectively richened in tumor sites and showed an excellent antitumor effect *in vivo*. The combination of PTT and BPFeP further improved the antitumor outcome. As a result, this study provided a platform to treat tumors by combining chemodynamic therapy and photothermal therapy to ablate solid tumors. The excellent photothermal property of BPFeP endowed such nano-system with on-demand antitumor ability, which ensured the spatiotemporal accuracy of cancer therapy.

## Supplementary Material

Supplemental Material
