# Supplementary material for: Construction of iron-mineralized black phosphorene nanosheet to combinate chemodynamic therapy and photothermal therapy
Source: Drug Deliv. 2022 Feb 17;29(1):624–36. doi: 10.1080/10717544.2022.2039810 (PMC8856058; doi:10.1080/10717544.2022.2039810)
Supplement: Supplemental Material [file IDRD_A_2039810_SM7782.docx]

**Supporting Information**

­­**Construction of iron-mineralized black phosphorene nanosheet to combinate chemodynamic therapy and photothermal therapy**

Zhaoqing Shi ^a^ , Jing Tang ^a^ , Chuchu Lin ^a^ , Ting Chen ^a^ , Fan Zhang ^b^, Yuxing Huang ^c^, Ping Luan ^d^, Zhuo Xin ^c^, Qianqian Li^e*^ and Lin Mei ^a,b*^

*^a^ School of Pharmaceutical Sciences (Shenzhen), Sun Yat-sen University, Shenzhen 518107, China*

*^b^ Tianjin Key Laboratory of Biomedical Materials, Key Laboratory of Biomaterials and Nanotechnology for Cancer Immunotherapy, Institute of Biomedical Engineering, Chinese Academy of Medical Sciences and Peking Union Medical College, Tianjin, 300192 China*

*^c^ School of Material Science and Engineering and Institute for Advanced Study, Nanchang University, Nanchang 330031, China*

*^d^ Guangdong Second Provincial General Hospital &Health Science Center, Shenzhen University, Shenzhen 518060, China*

*^e^Shenzhen Bay Laboratory, Shenzhen 518055, China*

* Corresponding author.

E-mail address: *meilin@bme.pumc.edu.cn (L. Mei); liqq_1993@163.com (QQ, Li)*


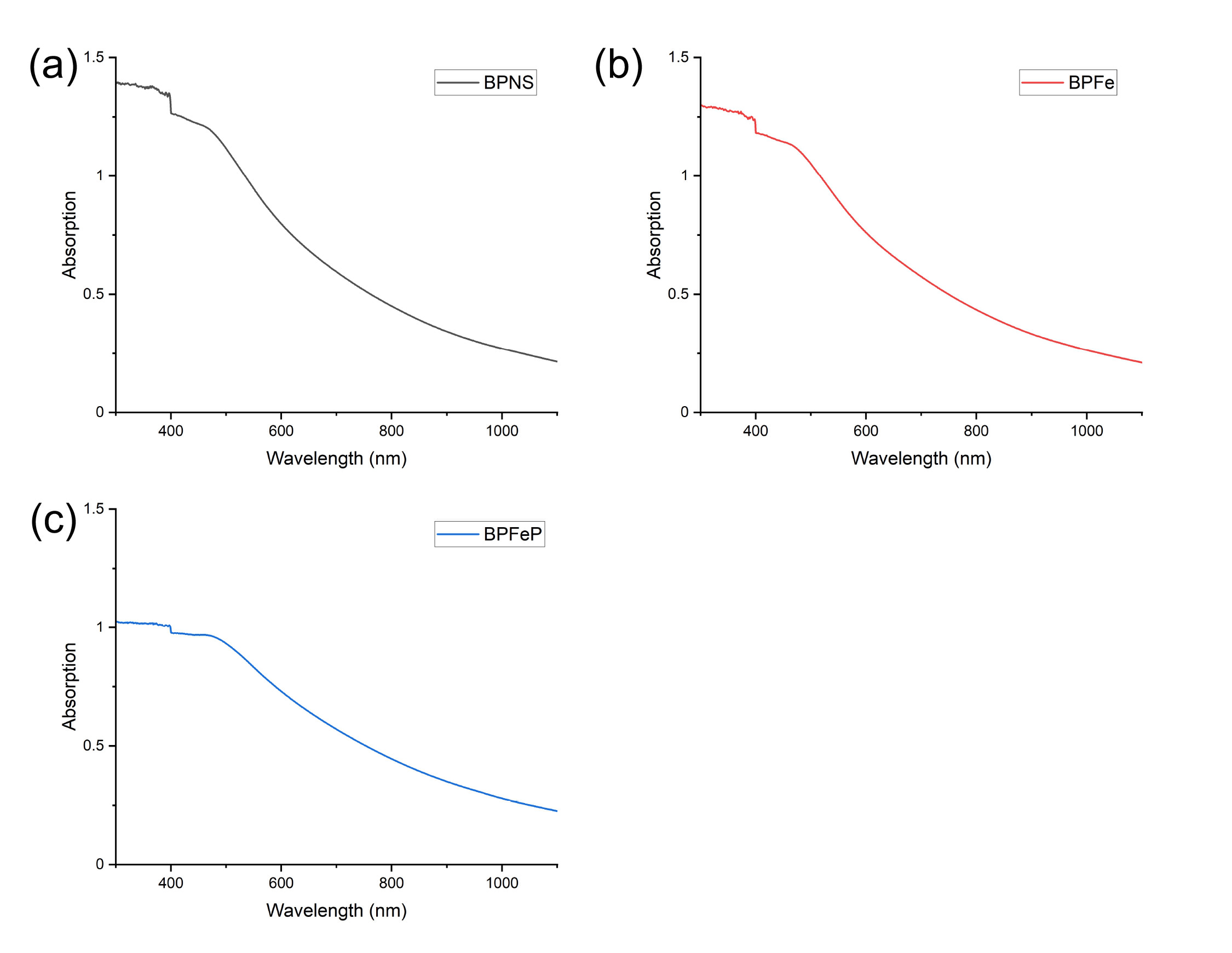


**Fig. S1.** The UV-vis spectra of BPNS, BPFe and BPFeP.


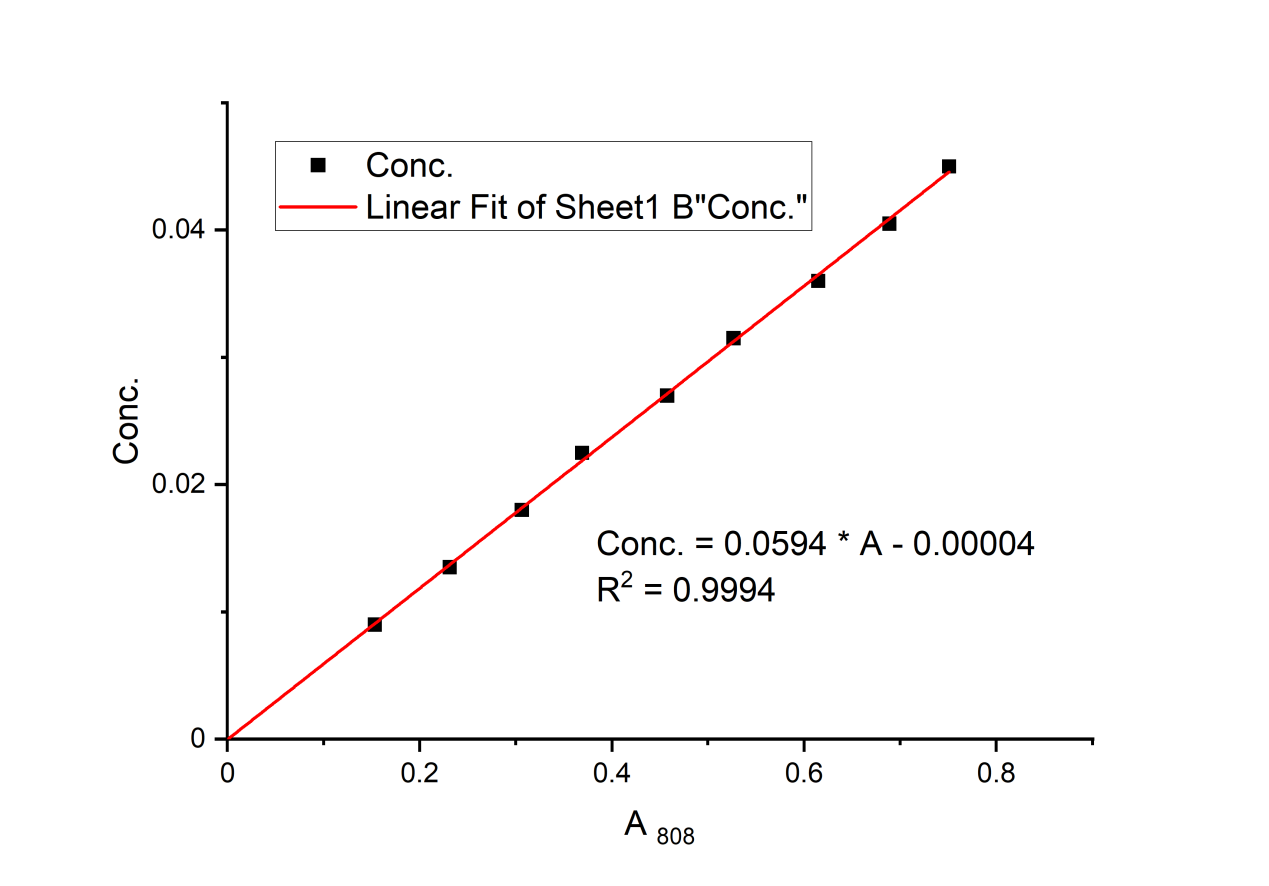


**Fig. S2.** The linear relationship of BPFe between concentration and optical absorbance at 808 nm.


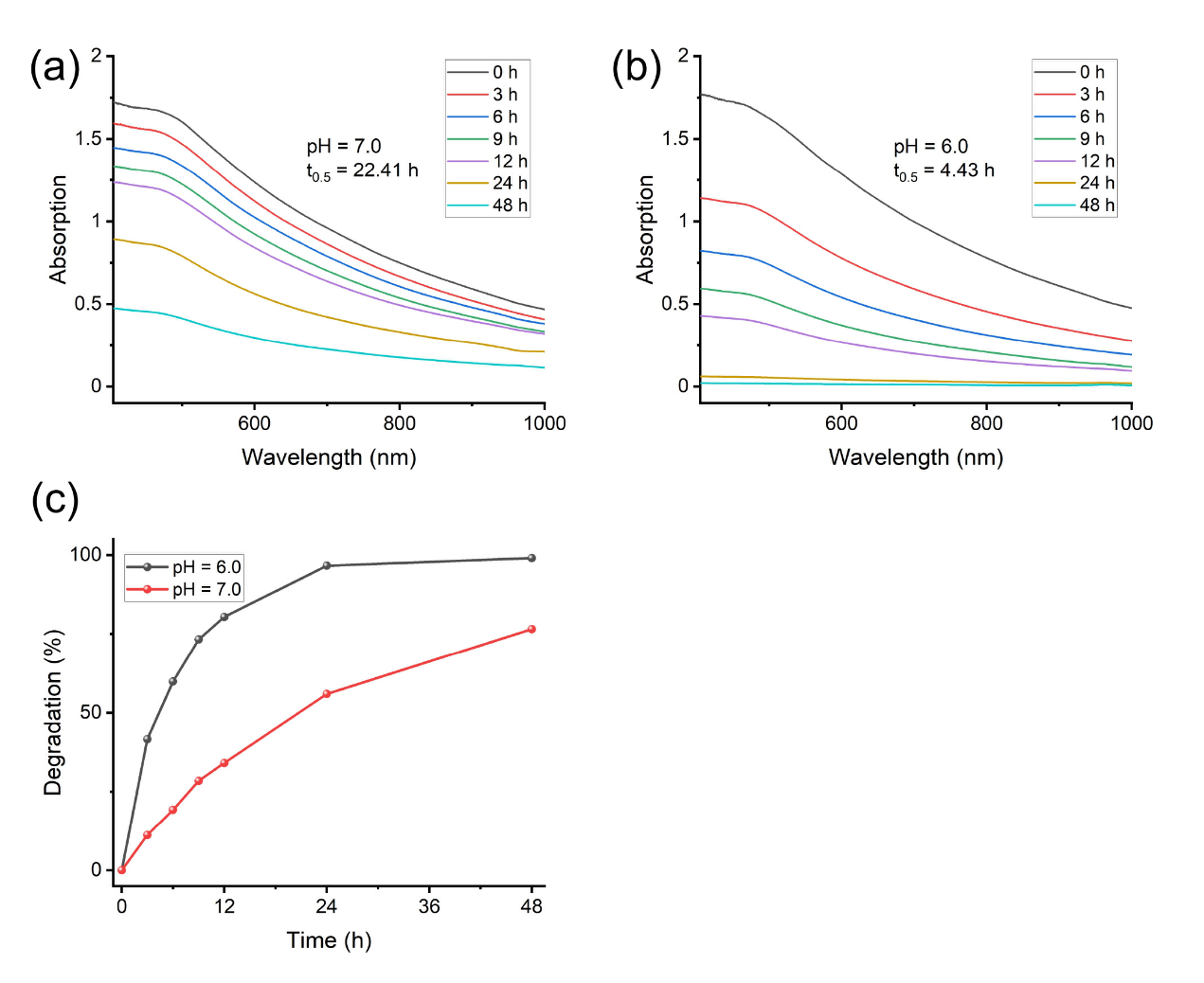


**Fig. S3.** BPFe showed faster degradation behavior in acidic pH environment compared with neutral pH environment. (a) Absorption spectrum of BPFe at indicated time in pH 7.0 PBS. (b) Absorption spectrum of BPFe at indicated time in pH 6.0 PBS. (c) Degradation behavior of BPFe in PBS of different pH.


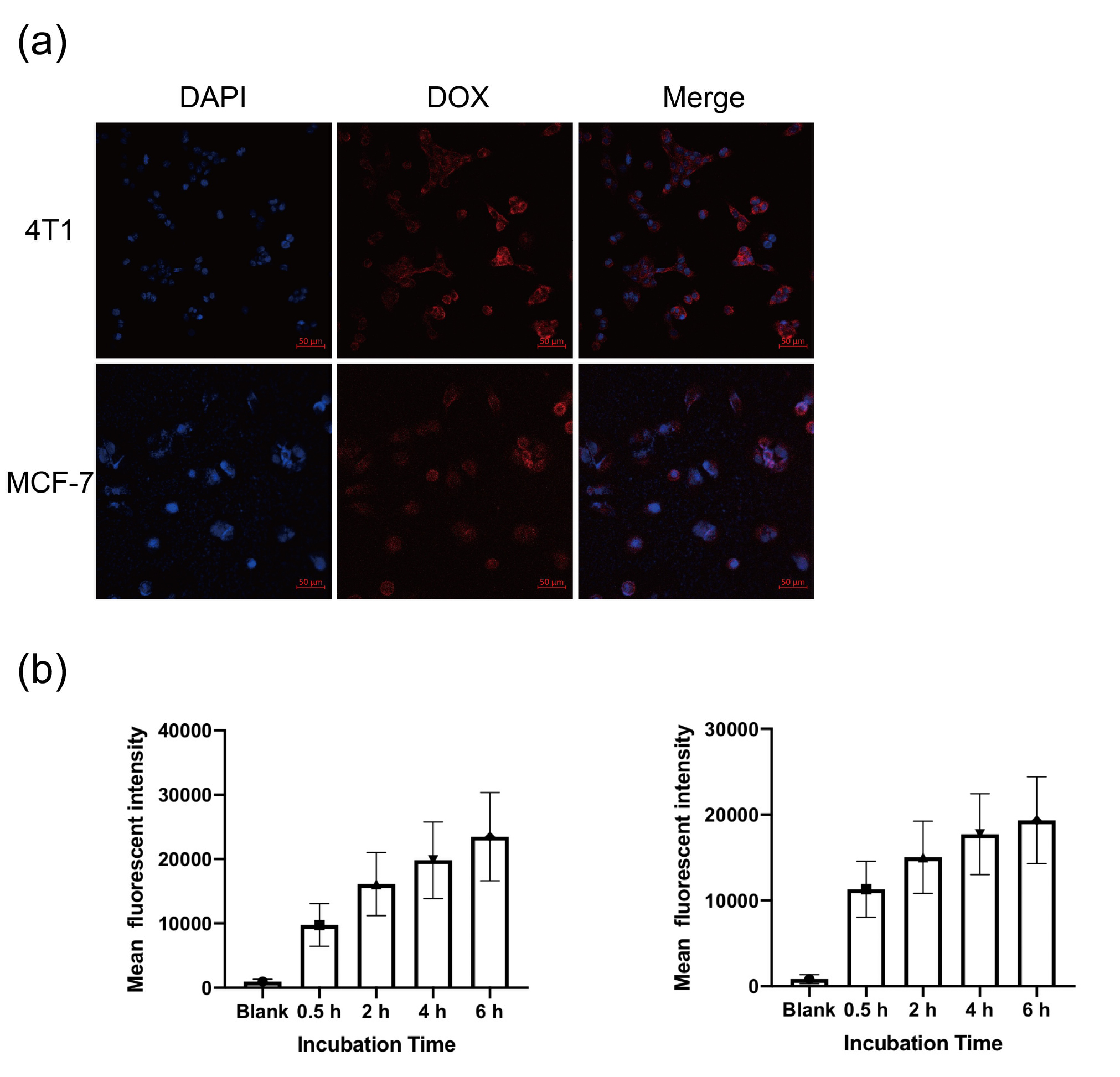


**Fig. S4.** The cellular uptake evaluation of BPFe. (a) CLSM image of 4T1 and MCF-7 cells after incubated with BPFe@DOX for 4 h. (b) FCM of 4T1 and MCF-7 after incubated with BPFe@DOX for indicated time.


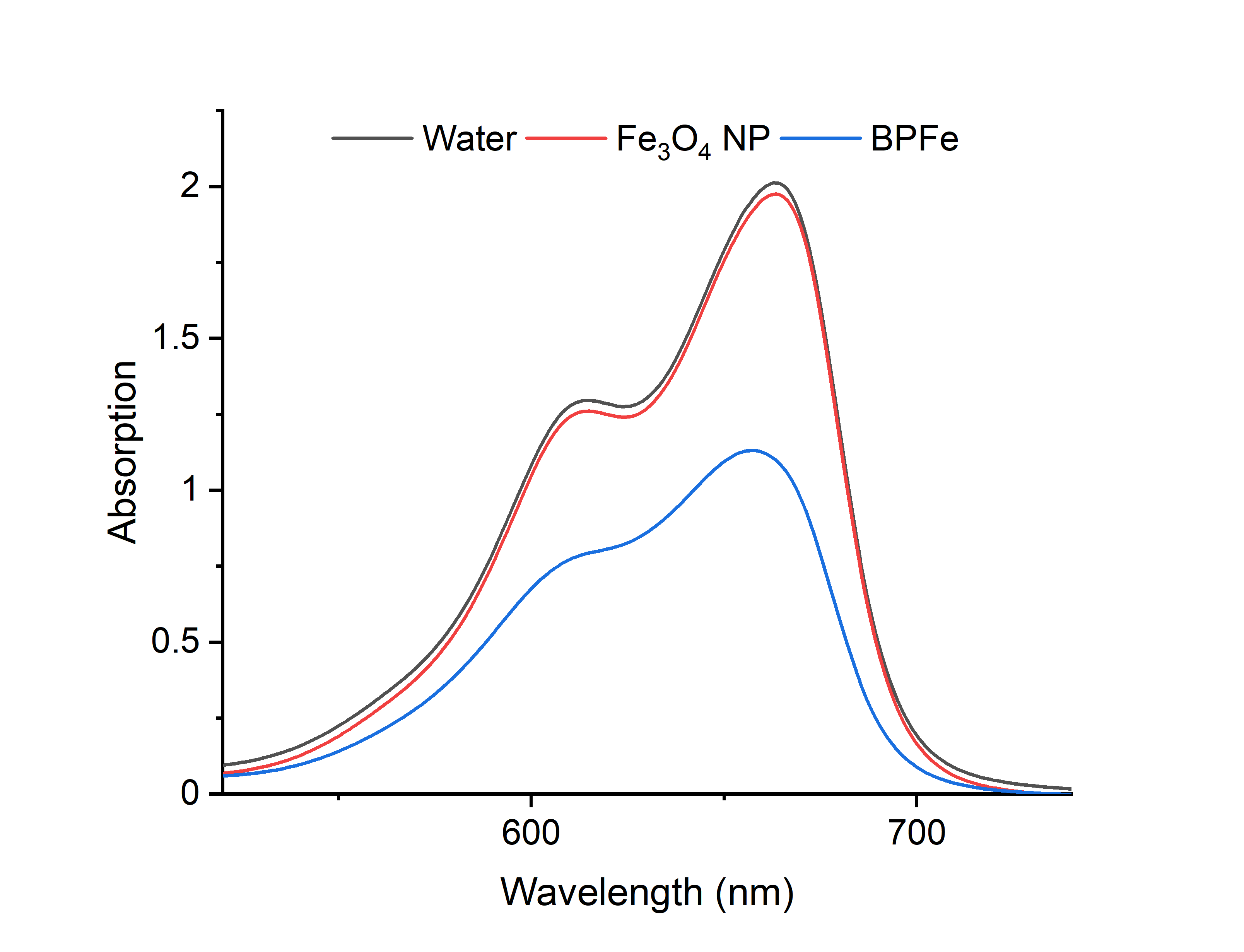


**Fig. S5.** Absorption spectrum of MB after treated with BPFe and Fe_3_O_4_@PEI, a commercial iron oxide nanoparticle. Fe mass concentration was 5 μg/mL.


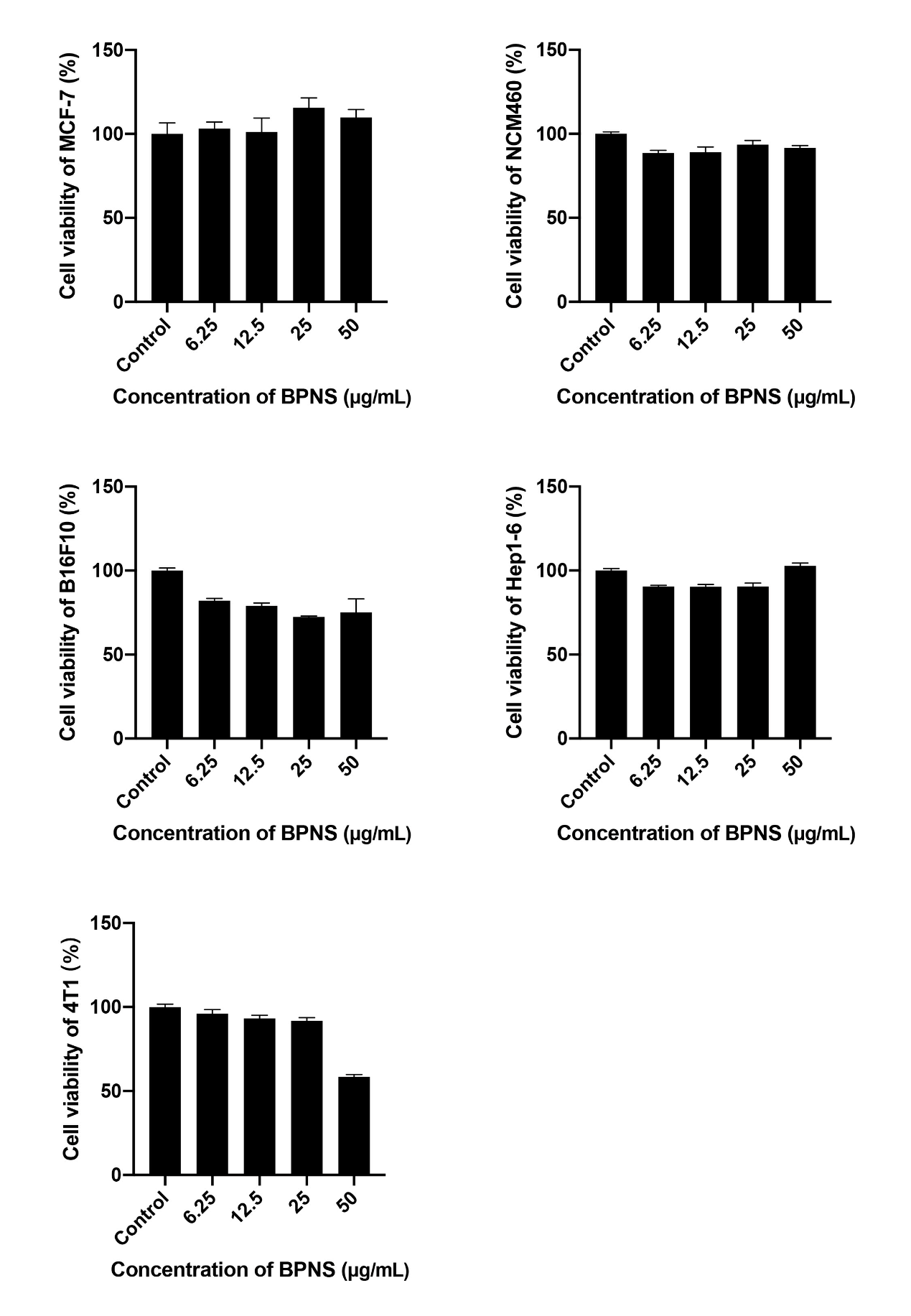


**Fig. S6.** BPNS rarely generated ⋅OH compared with BPFe. (a) Absorption spectrum of MB after treated with indicated concentration of BPNS. (b) The absorption at 660 nm variation of MB after treated with indicated concentration of BPNS or BPFe.


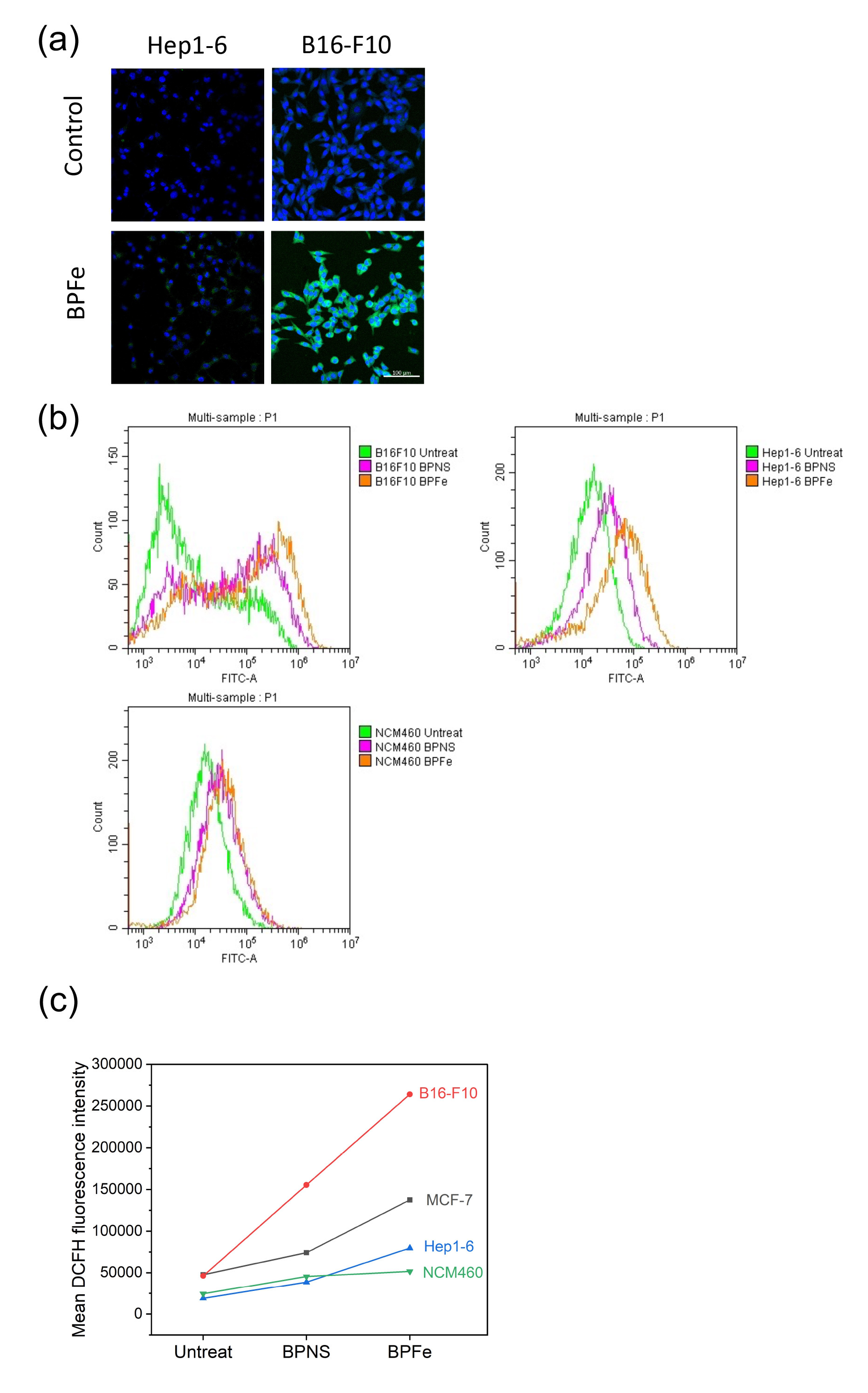


**Fig. S7.** The intracellular ROS assays. (a) CLSM images of BPFe treated B16-F10 cell lines, the green channel indicated the DCFH fluorescence. (b) FCM analysis of intracellular ROS in different cell lines after treated with BPNS or BPFe. (c) Mean DCFH fluorescence intensity of intracellular ROS assays.


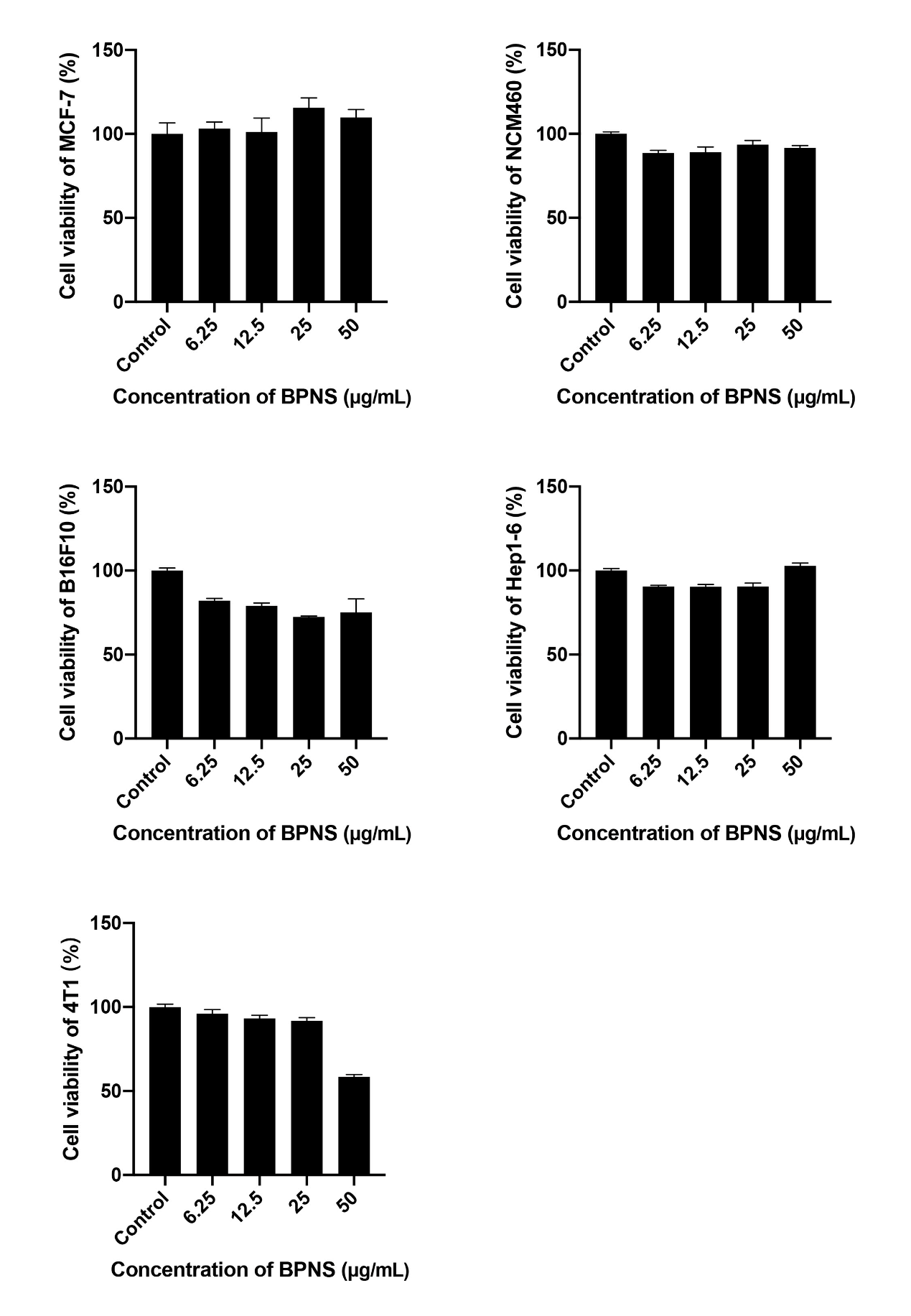


**Fig. S8.** Cytotoxicity of BPNS to the selected cell lines. The pure BPNS did not show obvious toxicity in these cell lines.


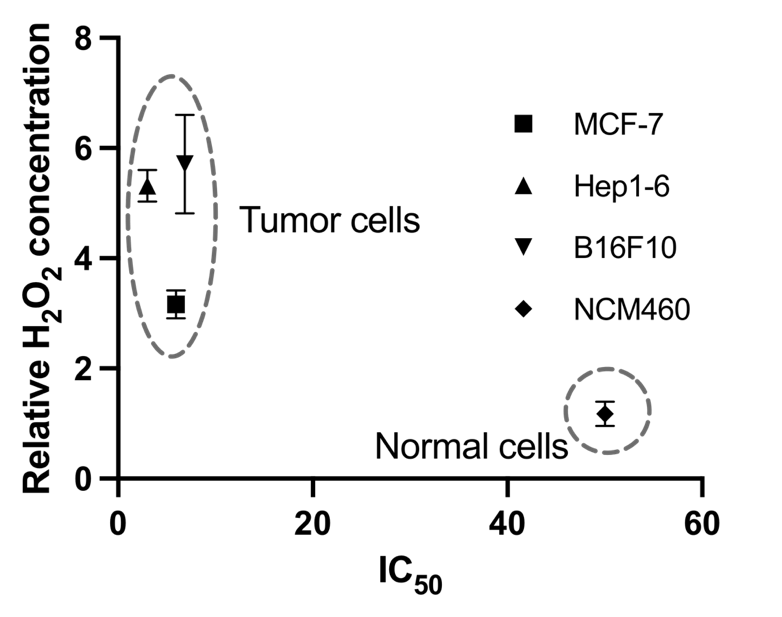


**Fig. S9.** Relationship between H_2_O_2_ level and IC_50_.


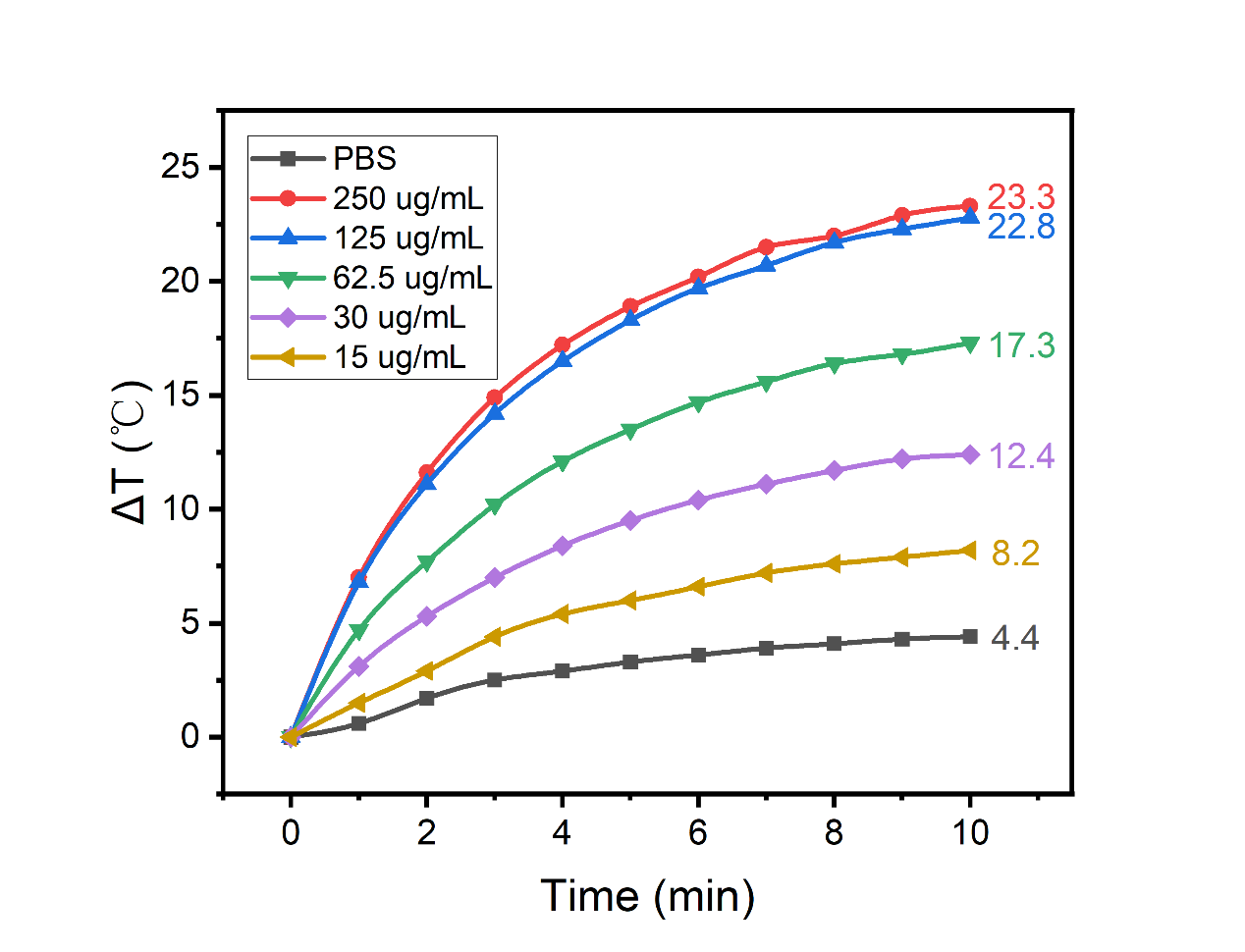


**Fig. S10.** The photothermal properties of BPNS.


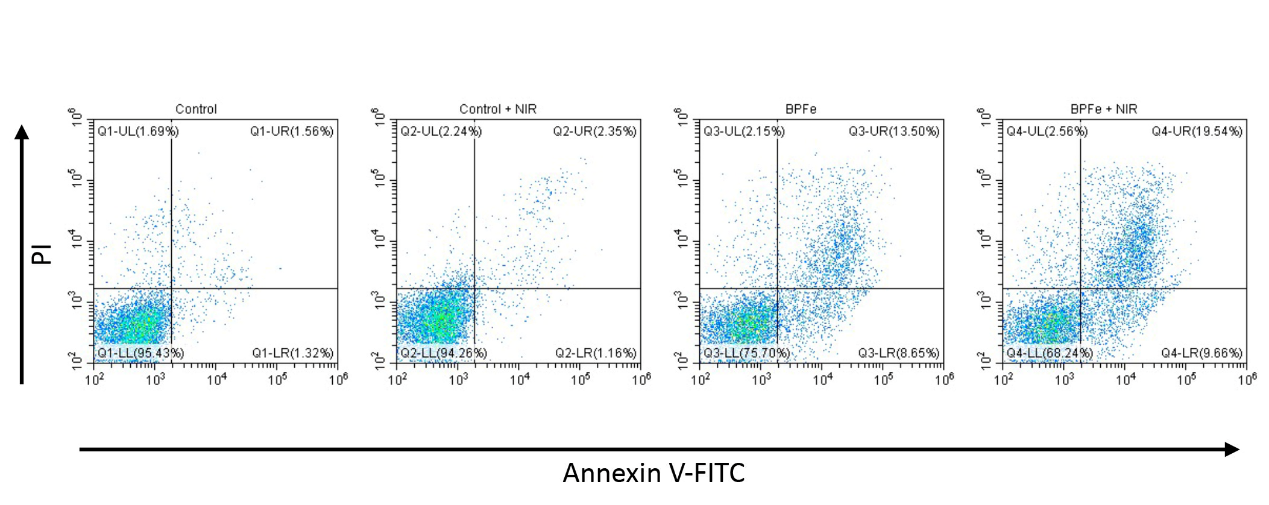


**Fig. S11.** The apoptosis assays on MCF-7.


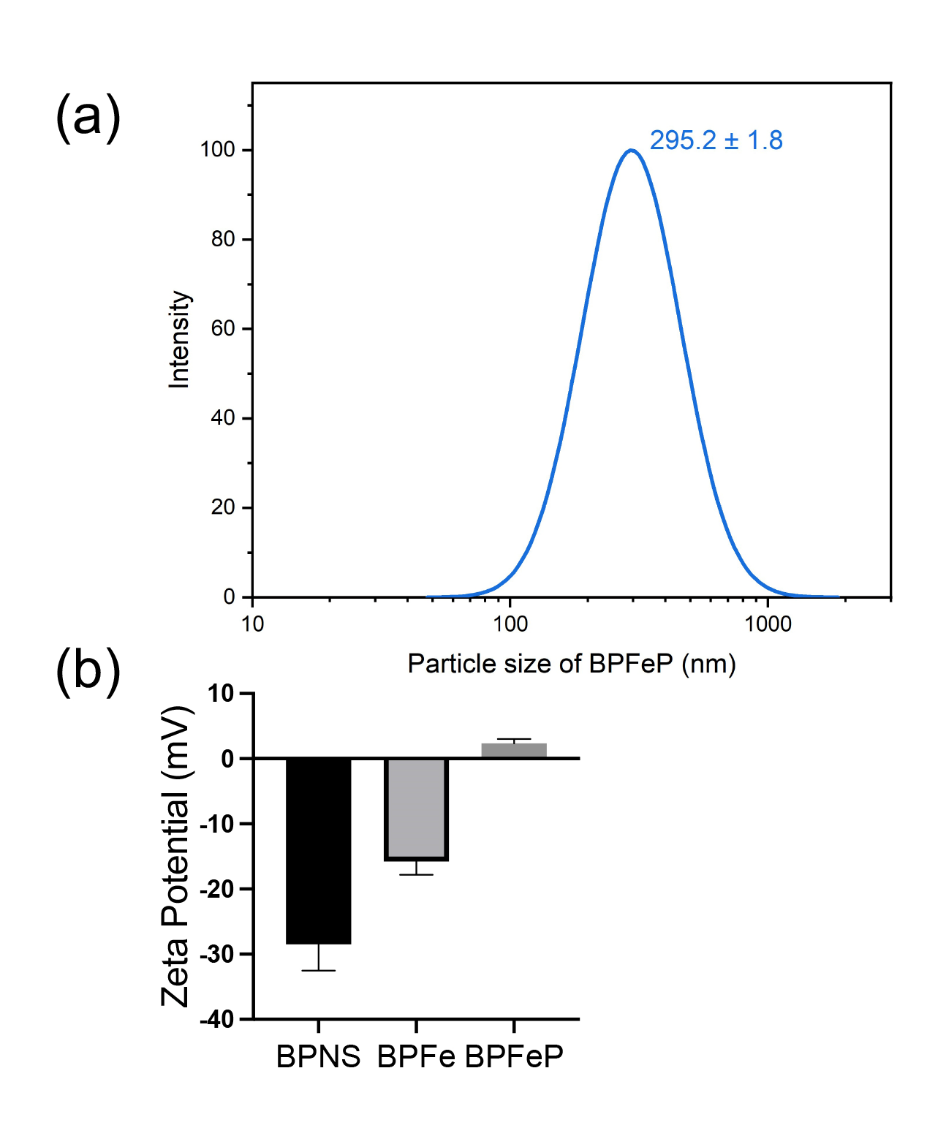


**Fig. S12.** (a) Particle size distribution of BPFeP. (b) Zeta potential of BPNS, BPFe and BPFeP.


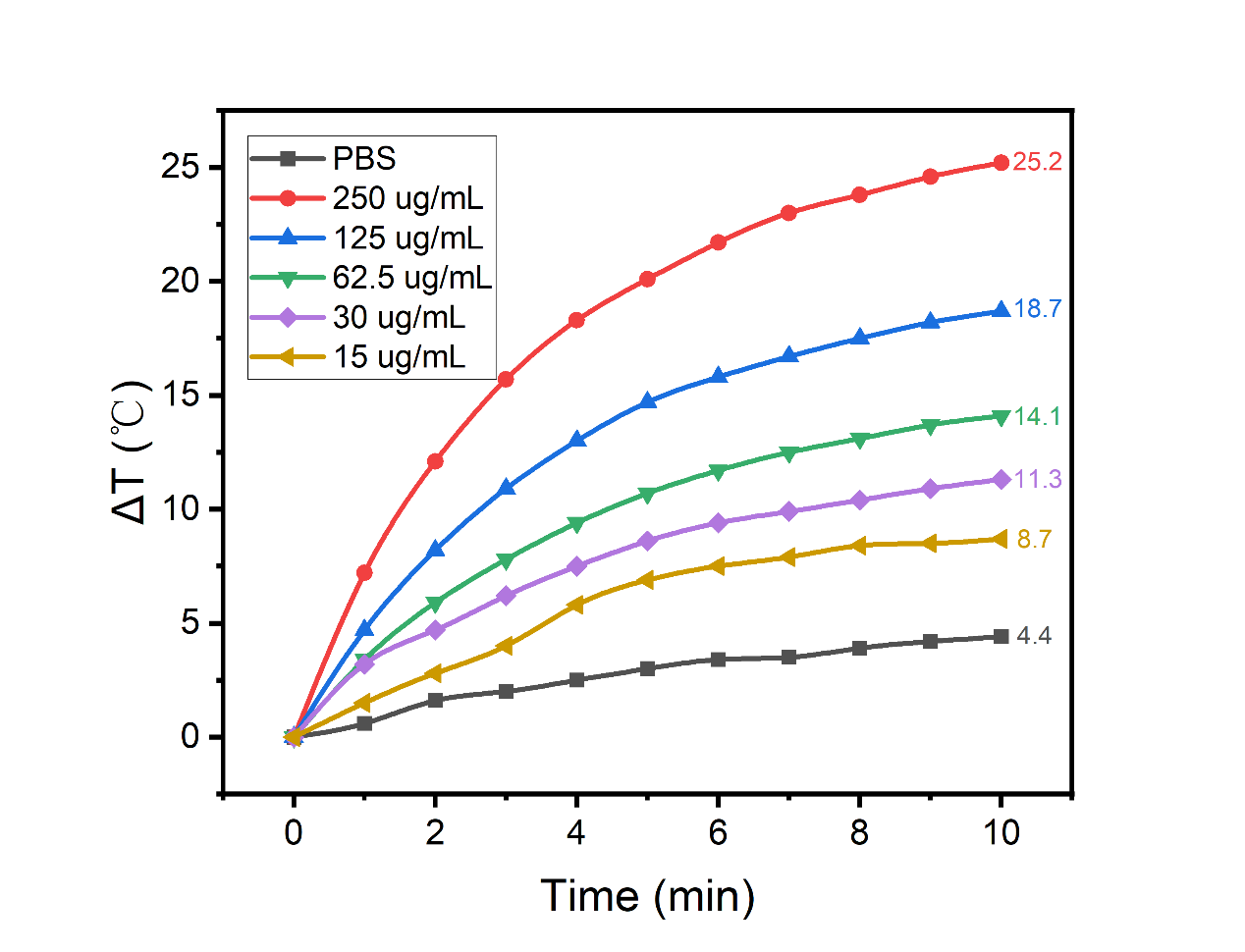


**Fig. S13.** The photothermal properties of BPFeP.


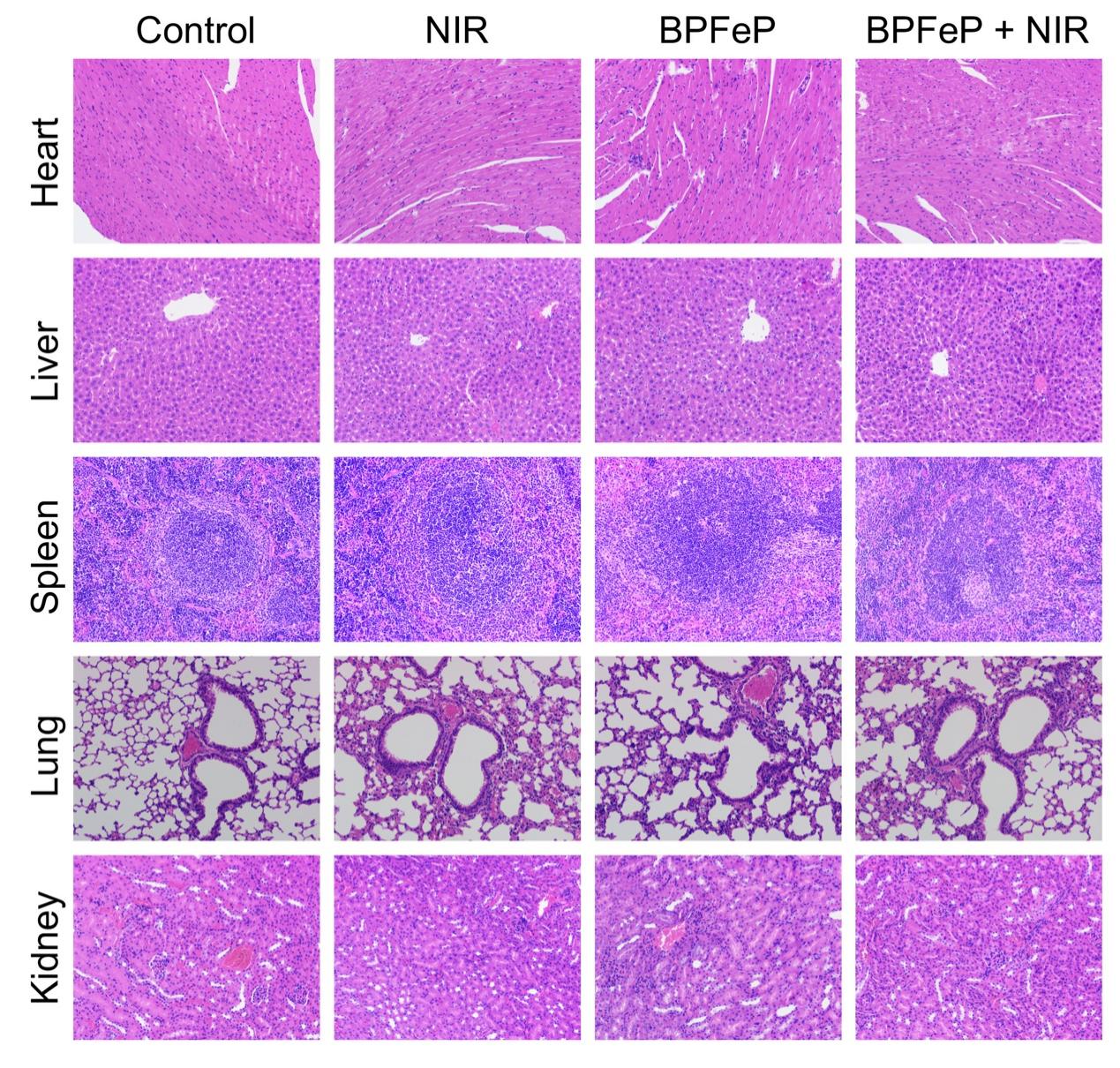


**Fig. S14.** The H&E staining images of excised slices major organs.

**Table S1.** Atomic analysis of BPFe according to XPS survey.

| Type of scan | Species | Peak binding energy (eV) | Atomic % |
| --- | --- | --- | --- |
| Fe 2p scan | Fe (II) | 709.98 | 9.25 |
|  | Fe (III) | 715.18 | 5.55 |
| P 2p scan | P (elemental) | 129.88 | 15.57 |
|  | P (elemental) | 130.68 | 8.17 |
|  | P-O (phosphorylated) | 133.68 | 61.45 |
